# Reference-free cell mixture adjustments in analysis of DNA methylation data

**DOI:** 10.1093/bioinformatics/btu029

**Published:** 2014-01-21

**Authors:** Eugene Andres Houseman, John Molitor, Carmen J. Marsit

**Affiliations:** ^1^School of Biological and Population Health Sciences, College of Public Health and Human Sciences, Oregon State University, Corvallis, OR 97331, USA and ^2^Section of Biostatistics and Epidemiology, Department of Community and Family Medicine, Geisel School of Medicine at Dartmouth, Hanover, NH 03755, USA

## Abstract

**Motivation:** Recently there has been increasing interest in the effects of cell mixture on the measurement of DNA methylation, specifically the extent to which small perturbations in cell mixture proportions can register as changes in DNA methylation. A recently published set of statistical methods exploits this association to infer changes in cell mixture proportions, and these methods are presently being applied to adjust for cell mixture effect in the context of epigenome-wide association studies. However, these adjustments require the existence of reference datasets, which may be laborious or expensive to collect. For some tissues such as placenta, saliva, adipose or tumor tissue, the relevant underlying cell types may not be known.

**Results:** We propose a method for conducting epigenome-wide association studies analysis when a reference dataset is unavailable, including a bootstrap method for estimating standard errors. We demonstrate via simulation study and several real data analyses that our proposed method can perform as well as or better than methods that make explicit use of reference datasets. In particular, it may adjust for detailed cell type differences that may be unavailable even in existing reference datasets.

**Availability and implementation:** Software is available in the R package RefFreeEWAS. Data for three of four examples were obtained from Gene Expression Omnibus (GEO), accession numbers GSE37008, GSE42861 and GSE30601, while reference data were obtained from GEO accession number GSE39981.

**Contact:** andres.houseman@oregonstate.edu

**Supplementary information:** Supplementary data are available at *Bioinformatics* online.

## 1 INTRODUCTION

Recently there has been increasing interest in the effects of cell mixture on the measurement of DNA methylation, specifically the extent to which small perturbations in cell mixture proportions can register as changes in DNA methylation (Adalsteinsson *et al.*, 2012; Bock, 2012; Houseman *et al.*, 2012; Lam *et al.*, 2012; Liu *et al.*, 2013; Reinius *et al.*, 2012). DNA methylation, tightly associated with alterations in the nucleosome DNA scaffold (and hence chromatin), is in part responsible for coordination of gene expression in individual cells (Ji *et al.*, 2011; Khavari *et al.*, 2011; Natoli, 2011). It is now appreciated that differentially methylated DNA regions (DMRs) distinguish cell lineages with high sensitivity and specificity (Baron *et al.*, 2006), and considerable research is now underway to delineate precise DMRs that define and specify a particular cell lineage. A recently published set of statistical methods exploits this association to infer changes in cell mixture proportions solely on the basis of a DNA methylation profile (Houseman *et al.*, 2012). These methods may broadly be conceived as the projection of DNA methylation data from a *target* dataset *S*_1_ onto a *reference* dataset *S*_0_ consisting of DNA methylation profiles for isolated cell types. For example, *S*_1_ may consist of DNA methylation measured in whole blood from a case–control study (Langevin *et al.,* 2012; Liu *et al.,* 2013; Koestler *et al.,* 2012; Wang *et al.,* 2013), while *S*_0_ may consist of corresponding DNA methylation profiles from isolated leukocyte cell types (e.g. CD4+ leukocytes, B cell lymphocytes, granulocytes) as provided by Houseman *et al.* (2012) and Reinius *et al.* (2012). Recently, some investigators have used these methods to adjust for cell mixture effect in the context of epigenome-wide association studies (EWAS, Liu *et al.,* 2013). However, these adjustments require the existence of reference datasets *S*_0_, and these sets may be laborious or expensive to collect. In addition, for some tissues such as placenta, saliva, adipose or tumor tissue, the relevant underlying cell types may not be known. In this article, we propose a method for conducting EWAS analysis when a reference dataset is unavailable, demonstrating that it can perform as well as the methods that make explicit use of a reference dataset.

## 2 STATISTICAL METHODS

Our proposed method, closely related to surrogate variable analysis (SVA, Leek and Storey, 2007), relies on a simple projection based on singular value decomposition (SVD), as does SVA. In SVA, the residuals of a linear model are decomposed into a factor-analytic structure and the factors are used subsequently in a regression model, with iteration resulting in a final set of surrogate variables. Our approach, which does not require iteration to obtain estimates, includes *unadjusted* linear coefficient estimates as columns of the matrix to be decomposed. As we demonstrate later in the text, this construction associates the residuals of the unadjusted model with the unadjusted coefficient estimates in a manner consistent with a linear mixing assumption.

We assume DNA methylation array results 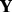, an 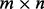 matrix representing DNA methylation measurements for *m* CpG sites and *n* subjects. We assume that the measurements are on a ‘beta value’ scale, having an interpretation as estimates of the proportion of methylated molecules corresponding to a given locus. In addition, we assume an 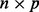 matrix 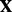 of covariates, including an intercept (in the first column), the phenotype of interest and potential confounders. The standard *unadjusted* EWAS analysis (on beta values) posits the linear model
(1)
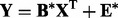

where 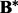 is an 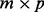 matrix of coefficients and 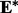 is an 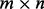 matrix of errors. Generally, analysis proceeds as if the rows of 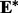 were independent, although permutation-based inference is sometimes used to account for potential correlation among rows, and 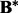 can sometimes include ‘surrogate variable’ confounders (Leek and Storey, 2007; Teschendorff *et al.*, 2011) that account for technical error. However, DNA methylation effects may be mediated through covariate effects on cell mixtures, i.e. 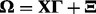 and 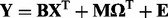, where 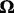 is an 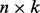 matrix of subject-specific cell proportions for *k* cell types (with rows summing to values 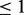), 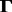 is a 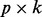 coefficient matrix representing cell-proportion effects, 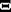 is an 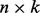 matrix of errors, 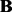 is an 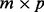 matrix of *direct* epigenetic effects (not mediated by effects on cell type), 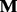 is an 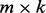 matrix of cell-specific mean methylation values (falling between 0 and 1) and 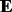 is an 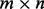 matrix of errors. The goal of an *adjusted* EWAS analysis is to estimate the direct effects 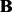. Note that 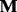 can be obtained from reference data, but it is unknown if a reference dataset does not exist. We explicitly posit a model on the beta-value scale because the effects are expected to be linear and additive only on this scale, not on the M-value scale often used (Liu *et al.*, 2013). Substitution results in the following linear model:
(2)
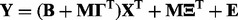

where it becomes evident that 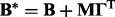 and 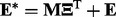. Although a naive analysis would treat the error matrix 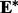 as having independent rows, an alternative model is to assume a factor-analytic structure on 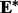 or 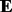, specifically
(3)
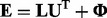

where 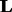 is an 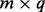 matrix of CpG-specific factor loadings, 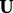 is an 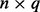 matrix of latent effects and 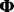 is an 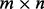 matrix of ‘uniqueness’ errors. This formulation is implicit in methods such as SVA (Leek and Storey, 2007) and independent surrogate variable analysis (ISVA, Teschendorff *et al.,* 2011), which are techniques proposed for addressing batch effects and confounders as well as cell mixture effects, although neither SVA nor ISVA explicitly posits this structure. Substituting (2) in (3) results in the following model:
(4)
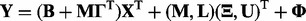

which expresses the explicit dependence of the latent structure of 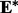 on the unknown cell-specific methylation matrix 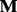. 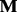 appears twice in (4), once in the error structure, and once as a random effect on 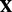. The SVA method extracts latent subject-specific effects using an SVD, which might also be used in this formulation as well, as long as the double appearance of 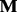 can be addressed. Careful inspection of (4) reveals that

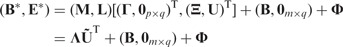

for some 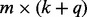 matrix 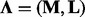 and 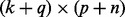 matrix 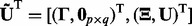, with 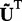 having at least *q* orthogonal columns. Although the *k* rows of the biologically determined matrix 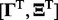 need not be orthogonal, the products 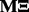 and 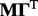 in (2) are not fully identified, in the sense that 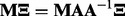 and 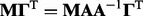 for any invertible 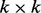 matrix **A**, including any that orthogonalizes the rows of 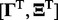; thus, it is possible to find a fully orthogonal 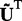 that still results in quantities that satisfy (2) and yield an identifiable 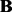.

Motivated by this observation, we propose applying an SVD on 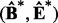 after fitting the unadjusted model (1); that is, we compute the SVD of the matrix obtained from the *unadjusted* model by concatenating the estimated coefficient matrix with the estimated residual matrix. Specifically, with dimension *d* fixed (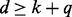), we find 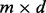 loading matrix 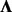, 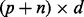 latent variable matrix 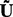 and 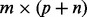 uniqueness error matrix 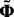 such that

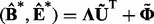

and 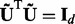. This is easily achieved by selecting the first *d* terms of the SVD of 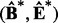, i.e. the SVD terms corresponding to the *d* largest singular values. Specifically, the SVD produces 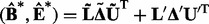, where 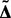 is a diagonal 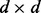 matrix, 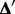 is a diagonal 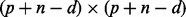 matrix, 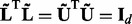, 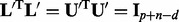 and every diagonal element of 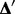 is less than every diagonal element of 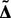; 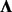 is thus obtained as 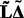. As 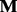 is a submatrix of 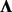, we propose the following estimator for 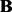, obtained as the residual of the projection of 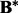 onto the column space of 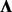:
(5)
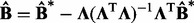



For any non-singular 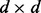 matrix 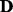,

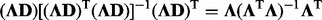

so that (5) is independent of the scales chosen for each column of 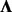. Although it is impossible to distinguish 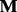 from 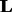 within 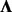, 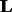 has an interpretation that is identical with the surrogate variables extracted by SVA, and that SVA would also be unable to distinguish 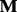 from 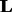.

Although this estimator is motivated by an explicit statistical model, its construction is somewhat *ad hoc*. Inference, therefore, demands an appropriate bootstrap estimate of coefficient standard errors. To adequately account for correlation in the error structure, we propose the following approach. First, 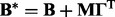 represents all systematic variation (cell-mediated and non-cell-mediated) and 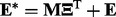 represents all unexplained variation (in both cell composition and non-cell-mediated variation), so that sampling with replacement from the columns of 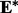 should form the basis of the bootstrap. However, the variance of the elements of 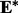 will depend on the corresponding elements of 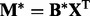, as the biologically determined values are approximately beta-distributed. [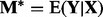 is a matrix of mean values 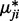, not the cell-specific methylation matrix 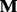.] As the variance of a beta-distributed variable with mean μ scales by 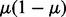, we obtain a matrix 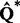 of mean-standardized errors by dividing each element 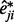 of 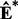 by 
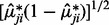
, where 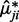 is the corresponding element of 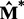. Thus, we construct bootstrap sample 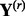 as 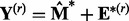, where the elements of 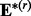 are obtained by first sampling with replacement from the columns of 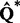, then multiplying the result, element-wise, by the elements 
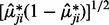
 (of the *un-resampled* matrix). This standardization approach preserves the relationship between the mean and variance of a beta distribution, while sampling column-wise preserves correlations across CpGs. As shown in the Supplementary Material, the latter property allows the bootstrap samples to form the basis of an omnibus test of significance over the entire array. Bootstrap estimates 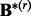 and 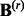 are thus obtained by fitting (1) on 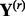, and subsequently recomputing (5). Standard errors for the elements of 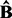 are obtained by calculating the corresponding standard deviations over bootstrap samples 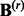, 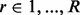 (with, e.g. *R* = 250 or *R* = 500). Bootstrap-based standard errors for 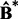 can be obtained simply by computing standard deviations from the bootstrap samples obtained by fitting (1) to the bootstrap samples 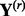.

The proposed methods can easily be adapted to accommodate missing values in 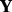 as follows: (i) rows of 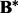 corresponding to rows of 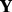 having missing values can be estimated on a row-by-row basis via complete-case analysis; (ii) the SVD producing 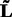 and 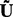 can be obtained from completely observed rows of 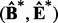; 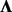 is then constructed from 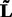 for completely observed rows, or else for rows with missing values by projecting 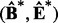 onto 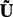 using a complete-case regression analysis. The bootstrap procedure introduces missing values by sampling from replacement from the columns of 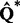, which will contain missing values for elements of 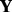 that are missing.

A remaining issue is appropriate selection of the dimension *d*. Teschendorff *et al.* (2011) propose a method for estimating the dimension of a latent surrogate variable using random matrix theory (RMT); application of their algorithm to the matrix 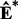 is one simple approach for estimating *d*. The simulation studies presented in the Supplementary Material suggest that this method performs well. As shown in the Supplementary Material, it outperforms a simple approach based on minimizing Akaike Information Criterion (AIC) and Bayesian Information Criterion (BIC), and data analysis results suggest that it produces estimates reasonably similar to those produced by the method suggested by Buja and Eyuboglu (1992) and implemented in the R package *sva*. In the case where 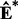 possesses missing values, only the completely observed rows are used.

Finally, we once again emphasize that the proposed methodology necessarily requires that analysis proceed on a linear (*β*-value) scale, because the logit-transform used to construct M-values destroys the linear mixing assumption. However, we acknowledge that a substantial portion of error may occur on the logit scale, owing to the fact that the individual probes used to interrogate the molecular methylation states are expected to have approximately lognormal distributions. We address this concern in the next section.

## 3 SIMULATION STUDY

We conducted a simulation study to confirm that the proposed methodology produces unbiased results. Simulation parameters were chosen to produce datasets as similar as possible to realistic DNA methylation datasets, although the dimensions were small enough to make the simulation study feasible. Fixing *m* = 1000, *n* = 250 and *k* = 4, we constructed 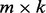 matrix 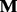 by selecting each element of the first 250 rows of 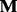 as a 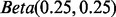 random variable, setting the remaining rows equal to zero; thus, the first 250 rows of 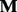 correspond to CpGs that are DMRs for *k* = 4 cell types. 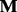 was held constant over all simulations. The 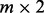 matrix 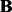 was constructed as 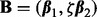, where 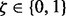 and 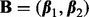 were generated (once for all simulated datasets) from a 3-part mixture model as described in the Supplementary Material, in such a way that non-zero direct effects tended to occur only for CpGs with mid-range values, and their signs tended to correlate inversely with the intercept. Details appear in the Supplementary Material (Section I), but for each simulated dataset there were 30 negative effects, 23 positive effects and 947 null effects. For each simulated dataset, the phenotype of interest, *x_i_* (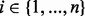), was generated as a 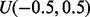 uniform random variable. Each row of the cell proportion matrix 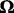 was generated as 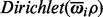, where the dispersion parameter 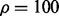 resulted in biologically plausible variation in cell proportions for a specimen such as blood, 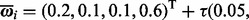 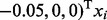, and 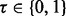 was a simulation parameter controlling the strength of the phenotype effect on cell mixture. Generation of the error component of the model was implemented in a manner that allowed us to investigate the effects of error on different scales, logit and linear. First, we presume that biological variation occurs on the linear scale, with error arising from a beta distribution. Thus, with 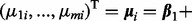 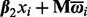, we generated beta-distributed values 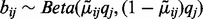, 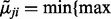 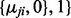, 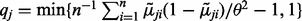 and 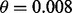; in other words, we chose row-specific dispersion parameters such that the average variance of *b_ji_* for row *j* over 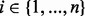 was about 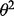. Simulations demonstrate relative insensitivity to θ, as shown in Supplementary Material (Section IV; Fig. S5(e)].

To obtain the measured methylation values, we added a *microarray error* of the factor-analytic form described by (3) but incorporated on a logit scale. Beta values *y_ji_* are typically constructed as the ratio 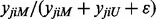, where *y_jiM_* is the measurement obtained from a probe designed to interrogate a methylated molecule, *y_jiU_* is the measurement obtained from the corresponding unmethylated probe and 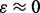 is a small constant chosen in advance. A common assumption in microarray analysis is that measurements such as *y_jiM_* and *y_jiU_* are lognormally distributed; consequently, 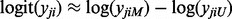 is normally distributed, and we expect the technical error introduced by the microarray to occur on the logit scale. For a latent error component on the logit (M-value) scale of measurement, we set *q* = 2 and generated the elements of the 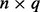 matrix 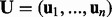 in (3) as standard normal variables, while the corresponding 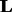 matrix was generated as 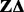, with the elements of the 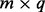 matrix 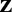 generated as standard normal variables and 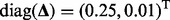. For the matrix 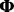 in (3), the elements of each row *j* were simulated as 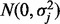, with 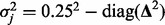 (i.e. the standard deviation of each value was 0.25, but the errors were correlated across CpGs). Thus, with microarray errors 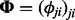, the simulated value *y_ji_* for row *j* and column *i* was generated as 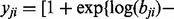 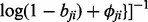. Table 1 describes the combinations of simulation parameters 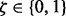 and 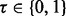 used for each of four scenarios used to investigate basic properties of proposed estimator. Supplementary Figure S2(b) provides a clustering heatmap displaying a typical dataset simulated under scenario #1. In each simulation (except those conducted to compare methods of dimension estimation), the RMT method of Teschendorff *et al.* (2011) was used to estimate the latent dimension.

**Table 1.** Simulation scenarios

Sim number	ζ	τ	Description
1	1	1	53 non-null direct eff., non-null cell mixture eff.
2	1	0	53 non-null direct eff., null cell mixture eff.
3	0	1	0 non-null direct eff., non-null cell mixture eff.
4	0	0	0 non-null direct eff., null cell mixture eff.

*Note*: Description of simulation parameters: ζ controls the strength of the direct (non-cell-mediated) effect on methylation; τ controls the strength of the effect of covariate on cell-mixtures.

The Supplementary Material describes additional simulations used to investigate several specific issues, as well as providing additional graphical results for the four main simulation scenarios reported here. Supplementary Material (Section III) reports the effects of larger sample sizes (*n* = 500). Section IV describes a simulation experiment designed to investigate the effect of different scales of error variability on the accuracy of different methods of dimension estimation (both in estimating the dimension itself and in the impact on Root-Mean-Squared-Error (RMSE)). Section IV also provides a comparison of our proposed methodology with SVA. Section V describes a simulation experiment designed to investigate power and Type I error. In every scenario considered, we simulated 100 separate datasets, and for each simulated dataset, we used 250 bootstrap samples for inference.

**Fig. 1.**
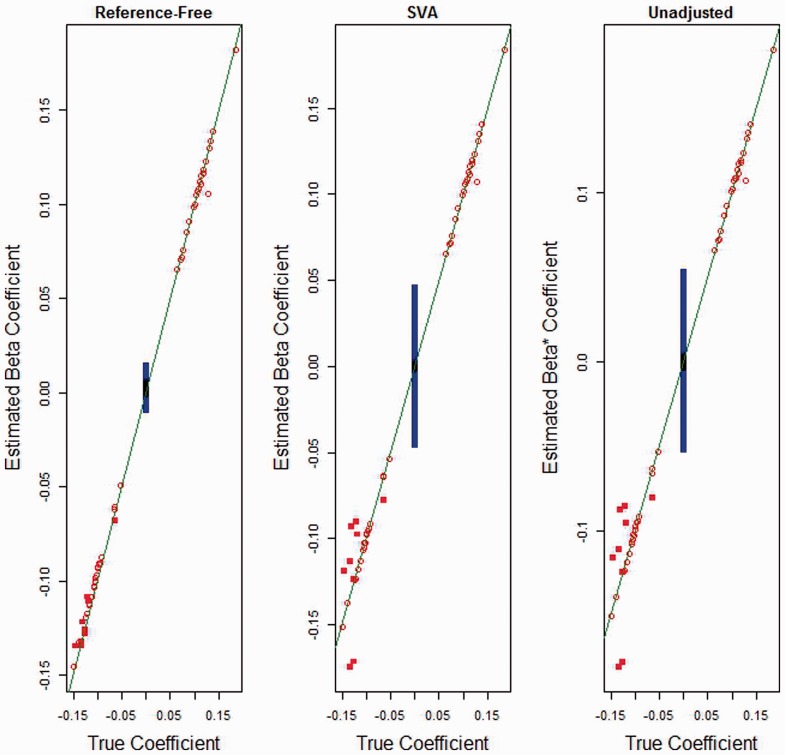
Simulation 1: estimated effect by true effect. Comparison of slope estimates: true direct effect (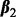) versus its estimate (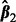), true direct effect versus the SVA-adjusted estimate and true direct effect (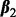) versus the unadjusted effect (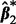). Squares indicate DMRs. Red indicates non-null CpGs. Black squares represent non-null DMRs

**Fig. 2.**
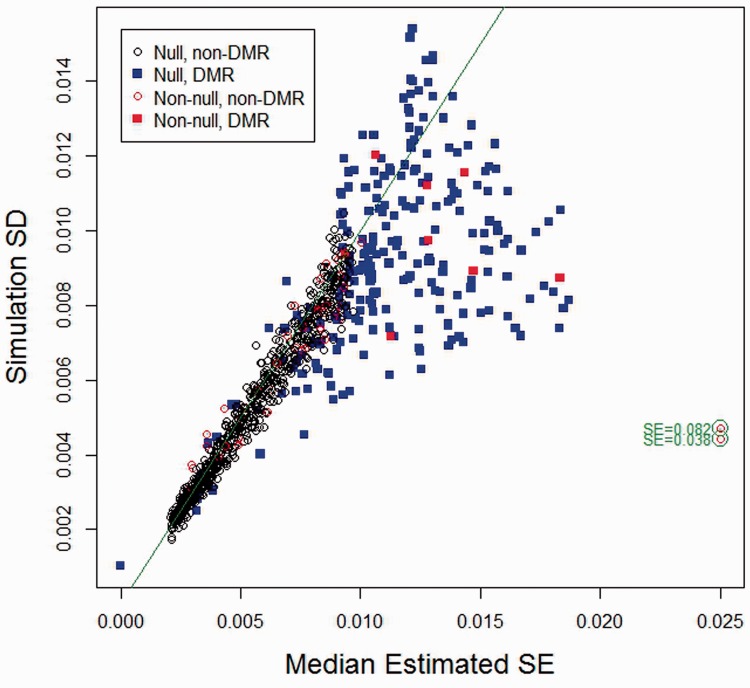
Simulation 1: bootstrap standard error by simulation standard deviation for 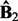. To increase legibility of the plot, SE estimates for two CpGs producing extreme bias have been moved to the left, as indicated

For simulation #1, Figure 1 compares slope estimates 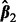 versus 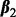, the SVA-adjusted variant of 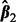 versus 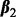 and 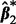 versus 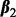, on each of the *m* = 1000 features. This figure demonstrates that although we expect the naive unadjusted estimator to provide an unbiased estimator of the total effect 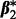, its estimates of direct effects are somewhat biased in comparison to our proposed estimator 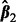, especially for the null slopes. It also demonstrates that SVA produces biases similar to the unadjusted analysis. This figure is consistent with Table 2, which reports the *total* RMSE [e.g. the square root of the simulation average of 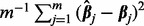] for each of the four comparisons across all four scenarios. Table 2 suggests similar behavior for null direct effects when the cell mixture effect is non-null (simulation # 3). When the mixture effect is null, 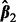 and 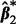 estimate 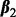 with about the same precision, as one would anticipate. Under simulation scenario #1, for each of 1000 features, Figure 2 plots simulation SD versus median bootstrap estimate (across 100 simulations) of the direct effect estimator. The bootstrap procedure appears tolerably unbiased, although our bootstrap standard error estimator yields apparently inflated estimates for some DMRs and a handful of non-DMR CpGs having non-null effect. The two very biased estimates result from intercepts lying near the zero boundary for mean methylation μ, resulting in non-linear effects (because of truncation) for some subjects having strongly negative values of *x*; in the Supplementary Material we provide evidence that the bias decreases in larger samples (Section III). Table 2 reports the median (over CpGs) of the ratio of median bootstrap standard error (over simulations) to simulation SD for all four scenarios, for both 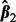 and 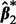, standard errors for the latter being computed using the standard linear model theory approach. Although the proposed bootstrap standard error methodology is imperfect, it appears to be as good as or better than the standard asymptotic methods used to compute standard errors for unadjusted effect estimates 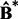. Supplementary Material (Section III) provides plots similar to those provided in Figures 1 and 2 for simulation scenarios #2, #3 and #4; they are consistent with the results and interpretations given here.

**Table 2.** Simulation results summary

Sim	RMSE				SE inflation	
	*BB*	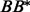	*BB_SV__A_*	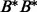	*B*	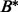
1	0.0077	0.0171	0.0148	0.0091	1.04	0.89
2	0.0063	0.0094	0.0054	0.0094	1.05	0.87
3	0.0070	0.0171	0.0150	0.0090	0.99	0.89
4	0.0054	0.0093	0.0053	0.0093	0.99	0.87

*Note*: BB, the RMSE for 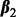 versus its estimate (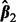); 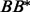, the RMSE for 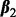 versus the unadjusted 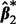; BB_SV A_,. the RMSE for 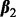 versus the SVA-adjusted variant of 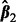; 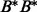, the RMSE for 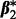 versus its estimate (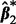, SE Inflation is the median (over all m CpGs) of 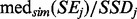, where the median SE is taken over simulations for each CpG j and similarly for the simulation standard deviation SSD.

The results of the simulation experiment described in Section IV suggest that the RMT method of dimension estimation has good accuracy in estimating the correct latent variable dimension when the magnitude of the latent effects is sufficiently large relative to other error components, superior to simpler methods based on AIC and BIC. RMT was able, in general, to estimate the dimension correctly even though the errors were incorporated on different scales (linear beta and logit M-value). In addition, the results suggest that when the microarray variation is relatively large, the reference-free and SVA methods have about the same level of error, presumably because the microarray error swamps the biological error. However, when the microarray error is smaller [but still consistent with realistic datasets, as shown in Supplementary Fig. S2(a)], our proposed reference-free method outperforms SVA, particular in estimating coefficients for DMRs; this latter phenomenon presumably occurs because the cell-mixture property is explicitly used in supervising the deconvolution. Section V describes a simulation experiment to investigate power and Type I error, using a bootstrap-based method for testing omnibus significance. It suggests appropriate Type I error control when 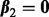, and reasonable power when the effects of 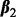 are large enough.

## 4 DATA EXAMPLES

**Fig. 3.**
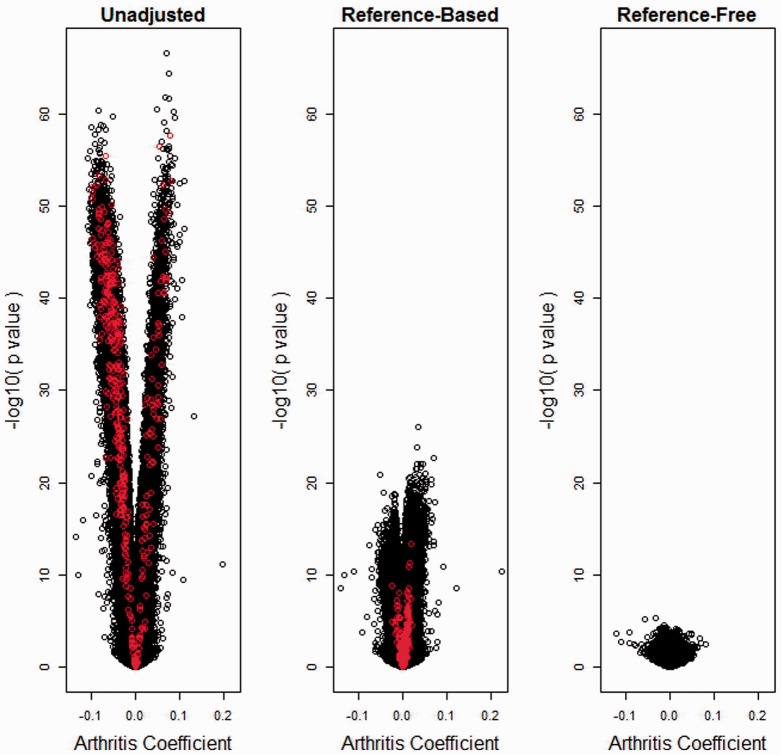
Arthritis dataset: volcano plots. Volcano plots for 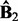 unadjusted for leukocyte composition, adjusted using the reference-based method that adjusts for six estimated cell type proportions and adjusted using the proposed reference-free method with *d* = 20. Red indicates 387 leukocyte DMRs (overlap between 450K array and 500 CpGs published by Houseman *et al.*, 2012)

**Fig. 4.**
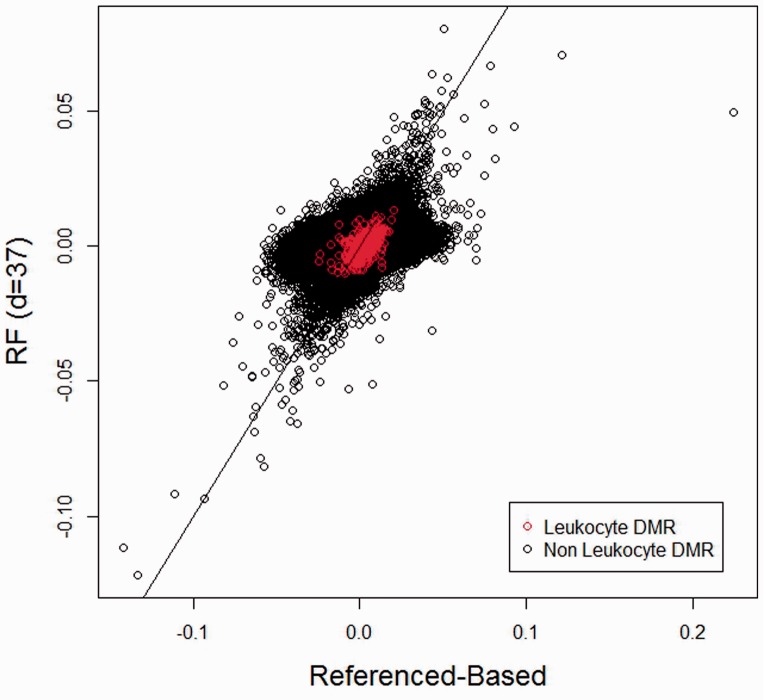
Arthritis dataset: reference-based versus reference-free. comparison of reference-free coefficient estimates 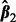 with the corresponding reference-based estimates

We demonstrate our proposed methodology on four datasets. The first consists of Illumina Infinium HumanMethylation450 BeadChip array data on which bisulfite-converted DNA from whole blood was hybridized; the data were obtained from Gene Expression Omnibus (GEO), Accession number GSE42861, and consisted of *n* = 689 subjects: 354 rheumatoid arthritis patients (cases) and 335 normal controls, originally published by Liu *et al.* (2013). Using the data available on GEO, we obtained four different estimates for the difference in DNA methylation measured on the beta scale between case and control at 384 410 autosomal CpGs whose Infinium probes contained no single nucleotide polymorphism (SNP) and had no SNP at a flanking G site (‘non-SNP CpG sites’). In the *unadjusted* analysis, we simply applied the *limma* procedure (Smyth, 2004), with design matrix consisting of an intercept and an indicator variable for case status. We then applied the method of Houseman *et al.* (2012) to data from 387 CpG sites overlapping between non-SNP CpG sites on the HumanMethylation450 and the 500 leukocyte differentially methylated regions made available publicly to infer leukocyte proportions (the full Illumina Infinium HumanMethylation27 dataset is available on GEO, Accession number GSE39981); in the *reference-based* analysis, we used limma to estimate case–control differences adjusted for leukocyte type by using five of the six available types: B-cell, CD4+ T, CD8+T, granulocyte and NK (monocyte proportions were dropped to avoid an ill-conditioned design matrix). In the *SVA-adjusted* approach, we used the R package *SVA* (version 3.6.0) both to compute the dimension of the surrogate variables and to determine the surrogate variables themselves. Using the method of Buja and Eyuboglu (1992) implemented in *sva*, we found *d* = 53 surrogate variables; after estimating the 53 surrogate variables, we adjusted for them using *limma* in a manner similar to the previous analysis. Finally, we applied our proposed *reference-free* analysis, with 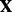 equal to the same design matrix used in the unadjusted analysis. In the latter analysis, the latent variable dimension was estimated to be 37 by the RMT method of Teschendorff *et al.* (2011); because simulations suggest accurate dimension estimation by the RMT method, we used *d* = 37. 500 bootstrap samples were used for inference. The DNA methylation dataset available on GEO contains no missing values. Figure 3 shows volcano plots of the arthritis case coefficient for the three different analyses, demonstrating diminished significant for both adjusted analyses shown in the figure (reference-based and reference-free). Interestingly, significance for the reference-free analysis is diminished relative to the reference-based analysis, suggesting that the six leukocyte types profiled by Houseman *et al.* (2012) and available in GEO Accession GSE39981 may be insufficient for analysis of blood data. This interpretation is further reinforced by Figure 4, which shows reference-free coefficient estimates by their corresponding reference-based estimates; there is general agreement between methods for coefficients with larger magnitude, except for a single CpG whose magnitude appears larger by the reference-based method than by the reference-free method; in general, for the relatively null CpGs, the reference-based method produces estimates of larger magnitude than the reference-free method. Supplementary Material (Section VI) provides volcano and scatter plots similar to those shown in Figures 3 and 4, but showing the SVA results. In addition, Supplementary Figure S9 shows a comparison of RMSE between our reference-free approach and SVA, where the 500-DMR reference-based analysis was used as a presumed gold standard. In general, the SVA results were dissimilar from both of the other adjusted analyses, with greater significance relative to both, and more similarity with the unadjusted analysis. This suggests inadequate adjustment by SVA. SVA results with *d* = 37 were similar to those obtained using *d* = 53 (data not shown in detail, but a summary appears in Supplementary Fig. S9). For each of the four analysis types, Table 3 summarizes overall significance as measured by *q*-value methodology (Storey *et al.*, 2004) implemented in the R package *qvalue*, including an estimate of 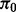, the proportion of nulls, as well as the number of CpG sites for which 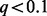. Interestingly, the unadjusted and reference-based analyses produced about the same estimated proportion of null CpGs as well as a relatively large number of CpGs for which 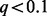. Despite apparently increased significance shown in the volcano plot appearing in the Supplementary Material, the SVA-adjusted approach produced a higher value of 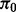 and fewer CpGs (though still a substantial number) for which 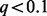. The reference-free analysis produced a much higher estimated value of 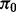 and no CpGs for which 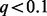. Although there were no significant *q*-values after reference-free adjustment, an omnibus test of significance proposed in Section V of the Supplementary Material results in an overall 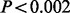 even after reference-free adjustment. As mentioned in Leek and Storey (2007), *q*-values can be misleading when applied to multiple *correlated* tests.

**Table 3.** Summary of significance for examples

Dataset	Number of CpGs	Analysis	Pr(null)	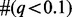
Blood (Arthritis)	3 84 410	Unadj	0.20	3 36 227
		Ref-based	0.24	3 12 133
		SVA-adj	0.41	2 07 569
		Ref-free	0.84	0
Placenta (SGA)	21 551	Unadj	0.43	3993
		SVA-adj	0.90	0
		Ref-free	0.92	0
Gastric (Tumor versus non-malignant)	26 486	Unadj	0.17	24 151
		SVA-adj	0.13	25 681
		Ref-free	0.71	1787

A possible explanation for decreased significance of the reference-free method relative to the other methods is that the reference-free approach led to less precise estimates. However, the median ratio of reference-free to reference-based standard error was 0.79, so that the reference-free approach led to estimates that were apparently more precise, overall, than the reference-based adjustment. Supplementary Material (Section VI) provides plots comparing estimates and standard errors for the three different methods. On the basis of these statistics and results, it appears that the reference-free method more precisely estimates effects that are smaller than those produced by the other two methods; thus, inflation of standard errors is an inadequate explanation. Another explanation for decreased significance of the reference-free method is that the reference-free approach potentially accounts for many more cell types than either the reference-based or reference-free approaches. The reference set provided by Houseman *et al.* (2012) differentiates granulocytes from other cell types, but does not differentiate neutrophils, basophils and eosinophils within the granulocyte category; it distinguishes CD4+ and CD8+ T-cells from other types of cells that are not T cell lymphocytes, but does not differentiate T helper cells, regulatory T cells or memory T cells. These types may be important differentiators of rheumatoid arthritis.

Our second analysis consists of array data for 92 independent peripheral blood mononuclear cell (PBMC) samples, assayed using the Illumina Infinium HumanMethylation27 (*27K*) technology, originally published in Lam *et al.* (2012) and available in GEO, Accession number GSE37008. For the purposes of this analysis, PBMC samples can be thought of as whole blood with granulocytes removed. In addition to DNA methylation data, complete blood count differential data were available for each sample, thus providing gold standard estimates for the fraction of the PBMC sample consisting of monocytes, assumed to be one minus the fraction of lymphocytes. Using several different approaches applied to the subset of autosomal CpGs, we examined the association between DNA methylation and the logarithm of il6 response to phorbol-12-myristate-13-acetatein (‘log pma’), a potent cell division promoter. The first analysis was unadjusted; the second analysis was adjusted for monocyte fraction; the third and fourth analyses were adjusted for blood cell fractions estimated using the approach of Houseman *et al.* (2012), similar to the approach described above, with the top 100 or 500 DMRs published in Houseman *et al.* (2012). Koestler *et al.* (2013) provide a detailed study of the application of reference-based estimates of cell proportion using this dataset. In the fifth approach, we adjusted for *d* = 11 surrogate variables using SVA, with *d* obtained by the method of Buja and Eyuboglu (1992). In the sixth approach, we applied the reference-free approach proposed in this article with *d* = 10, estimated via RMT. Both the third and fourth approaches produced similar results, and results reasonably similar to the second approach. The reference-free approach produced slightly increased significance over approaches 2–4. The SVA results were similar to the reference-free approach, although the resultant RMSE values were larger for the SVA approach than for the reference-free approach, when monocyte-adjustment or reference-based adjustment was used as a gold standard [Supplementary Fig. S10(o)]. The unadjusted analysis produced results that were substantially more significant than those produced by the other four approaches. Details of this analysis appear in Section VII of the Supplementary Material. In summary, our proposed reference-free approach produces results similar to (though slightly less variable than) those produced by SVA, to reference-based adjustments as well as adjustment by a known (though coarsely differentiated) gold standard, but quite distinct from the unadjusted approach.

**Fig. 5.**
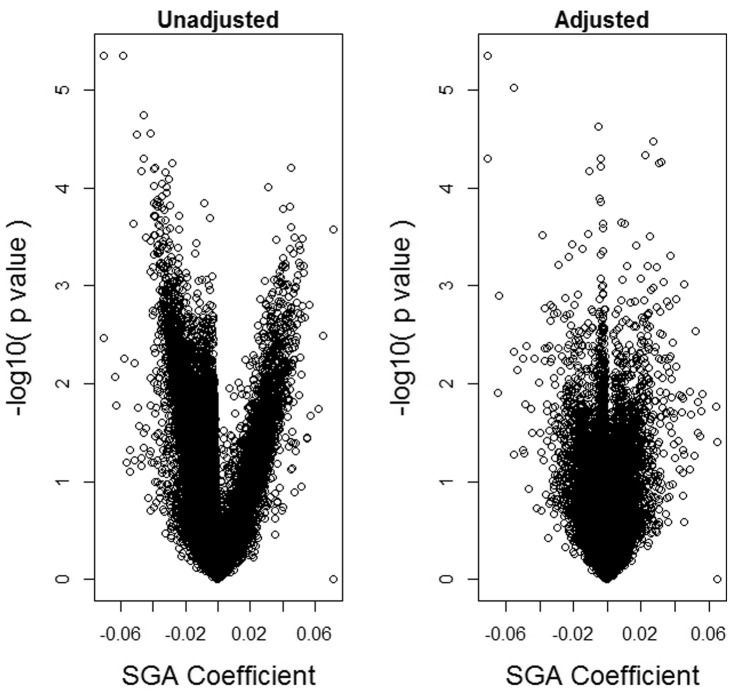
Placenta dataset: volcano plots. Volcano plots for 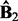 unadjusted for leukocyte composition and adjusted using the proposed reference-free method with *d* = 12

**Fig. 6.**
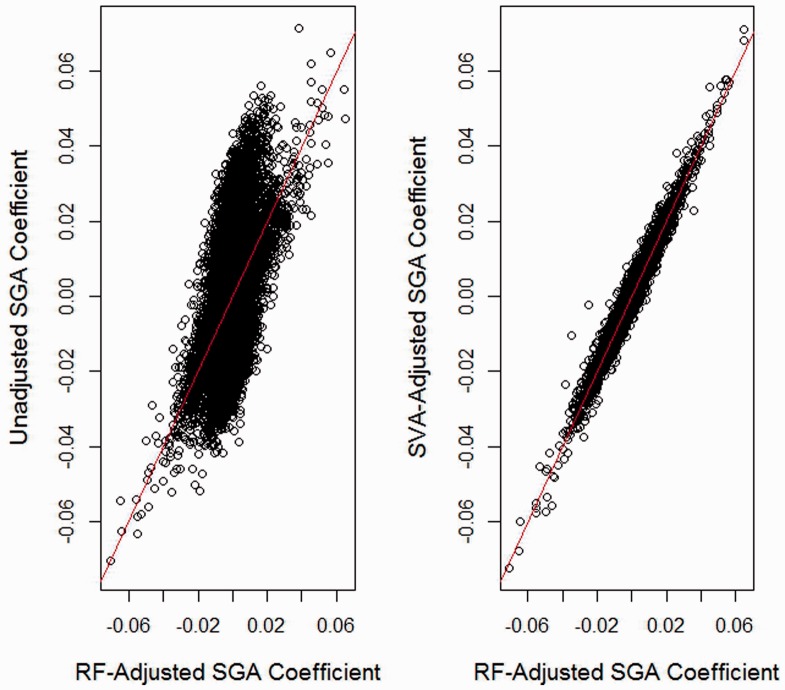
Placenta dataset: comparison of reference-free adjustment with unadjusted and SVA-Adjusted. Comparison of reference-free coefficient estimates 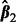 with the corresponding unadjusted and SVA-adjusted estimates

Our third analysis consists of 27K array data for 176 placenta samples originally published in Banister *et al.* (2011). All *n* = 176 infants considered in this analysis were of gestational age greater than 37 weeks. Data were adjusted for BeadChip effect using ComBat (Johnson *et al.*, 2007). Maternal-age adjusted differences in placental DNA methylation between 52 small-for-gestational age (SGA) infants and 124 normal infants at 21 551 autosomal non-SNP CpG loci were estimated using three methods. In the *unadjusted* analysis, *limma* was used with design matrix consisting of an intercept, an indicator variable for SGA, and maternal age. In the *SVA-adjusted* analysis, we additionally adjusted for *d* = 13 surrogate variables, with *d* obtained by the method of Buja and Eyuboglu (1992). In the *reference-free* analysis, we used our proposed method with *d* = 12 (estimated via RMT). The volcano plots shown in Figure 5 suggest that the SVA and reference-free analyses produce modestly diminished significance compared with the unadjusted analysis. Figure 6 shows adjusted effect estimates by their corresponding unadjusted estimates and by SVA-adjusted estimates; the figure suggests that a substantial fraction of CpGs demonstrates slightly larger effect magnitude when compared with their adjusted counterparts. As shown in Figures 5 and 6, as well as Supplementary Material (Section VIII), SVA and the reference-free methods produce almost identical results. In addition, Table 3 suggests diminished significance of results in the unadjusted analysis. Overall, the analysis suggests that much of the effect of SGA on DNA methylation may be explained by cell mixture, but that the mixture effect is substantially smaller than that occurring for DNA methylation measured in blood.

Our final analysis consisted of 27K data for 203 gastric tumors and 94 gastric non-malignant samples, originally published by Zouridis *et al.* (2012) and available in GEO, Accession number GSE30601. In each of the three analyses of DNA methylation at autosomal CpGs, we compared tumors to non-malignant samples. The first analysis was unadjusted; the second analysis was adjusted for 27 surrogate variables (with *d* = 27 determined by the method of Buja and Eyuboglu, 1992); in the third, we applied the reference-free approach proposed in this article, with 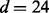, obtained via RMT. In general, the unadjusted, SVA and reference-free approaches produced different results. The SVA and reference-free approaches differed systematically (Wilcoxon 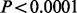) at CpGs mapped to polycomb target genes (Bracken *et al.*, 2006; Lee *et al.*, 2006; Schlesinger *et al.*, 2007; Squazzo *et al.*, 2006), with the reference-free approach demonstrating greater hypomethylation in tumors at PcG targets. Thus, compared with the other approaches, the reference-free method may demonstrate superiority in identifying biologically relevant alterations of DNA methylation in highly heterogeneous tumor samples. A summary of significance appears in Table 3; details of the analysis and graphical results appear in Supplementary Material (Section IX).

## 5 DISCUSSION AND CONCLUSIONS

We have proposed a *reference-free* method of conducting EWAS while adjusting for cell mixture. Following on work by Houseman *et al.* (2012), this method posits a statistical model that involves a latent variable representing mean methylation, together with a factor-analytic error model consistent with the surrogate variable approaches of Leek and Storey (2007) and Teschendorff *et al.* (2011). We have also proposed a companion bootstrap methodology for estimating standard errors.

Our simulation studies suggest that our proposed approach returns reasonably unbiased estimates of the *direct effect*, i.e. the effect not mediated by cell type, and reasonable standard error estimates. They also suggest adequate control of Type I error and reasonable power to detect direct effects of sufficient magnitude. Our simulations also suggest that the RMT method of dimension estimation, proposed by Teschendorff *et al.* (2011), performs well for estimating the dimension of the latent structure. Finally, our method performs about as well as surrogate variable analysis (SVA) when technical error is of large magnitude, and better than SVA when technical error is of smaller (but still realistic) magnitude. We have demonstrated our method on four separate datasets. The first was a rheumatoid arthritis dataset, where DNA methylation in blood shows substantial attenuation in effect after applying our reference-free approach, even in comparison to an approach similar to that used by Liu *et al.* (2013), wherein DNA methylation was adjusted for cell types profiled by Houseman *et al.* (2012) using methodology proposed in that paper. A possible explanation is that DNA methylation effects may be mediated by cell types not profiled by Houseman *et al.* (2012). The second set consisted of PBMC data and associated effects of il6 response to a potent tumor promoter, for which we demonstrate that our proposed reference-free approach produces results similar to reference-based approaches but dissimilar from an unadjusted analysis. The third set was a placental dataset for which no reference data were available for component cell types. In this dataset, effects of SGA showed modest attenuation after applying our reference-free method. This suggests that the effects of growth restriction on DNA methylation are likely less mediated by changes in cell distribution, a result that one might anticipate in a solid tissue sample such as placental tissue. The fourth analysis compared gastric tumors with non-malignant gastric tissue, showing differences in results between mixture-adjusted and unadjusted analyses, but with significant direct effects produced even in the adjusted analyses. In this last example, there is evidence that the results of the reference-free method may be more biologically meaningful than those produced by SVA.

An interesting aspect of our methodology is its tendency to produce ‘spikes’ in volcano plots, such as those shown in Figure 5 or Supplementary Figure S12(a). These are caused by shrinkage of standard errors, particularly at DMRs, i.e. CpGs that drive the cell mixtures in 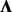. Because these CpGs collectively borrow statistical strength from each other, their standard errors tend to be smaller. This phenomenon is evident in Supplementary Figures S7(b) and S10(d), which compare reference-free and unadjusted standard errors for the blood and PBMC datasets, respectively; these plots demonstrate that for many known leukocyte DMRs, the standard errors in the reference-free method are noticeably smaller than the corresponding standard errors from the unadjusted analysis. The same phenomenon is evident in Supplementary Figure S7(h), which plots the standard errors from the reference-adjusted analysis against the corresponding standard errors from the unadjusted analysis, and demonstrates shrinkage of standard errors at leukocyte DMRs.

Unlike the SVA approaches of Leek and Storey (2007) and Teschendorff *et al.* (2011), we posit a specific data generation model that incorporates a factor-analytic structure that is implicit in the previously published methods. However, we incorporate *all* CpG features in the factor-analytic structure, rather than attempting to select a subset of features that are optimally informative. While there may be some loss of precision in using all CpG features present on a given array, we view this as an acceptable sacrifice to accommodate an agnostic approach that permits any CpG to serve as a DMR. In addition, the surrogate variables for which our model implicitly adjusts have an explicit mixing interpretation. Our simulations suggest equivalent or better results using our proposed method; the data analyses demonstrate that our method can produce results that are similar to SVA, as in the PBMC and placenta examples, or results that are distinct, as in the arthritis and gastric tumor examples. In the gastric tumor analysis, there is a suggestion that our reference-free approach may produce results slightly more consistent with known biology. The reference-free approach is able to do this without the somewhat time-consuming comparison of candidate surrogate variables with potential confounders; however, it achieves this result by essentially projecting unadjusted effect estimates on an error matrix, with a linearity assumption that, while biologically motivated, may sometimes fail. In addition, our method is designed to deconvolute cell mixtures; it is not designed to uncover confounders that are specific to technical sources of variation. Thus, SVA might be applied on an M-value scale to extract surrogate variables that are specific only for technical variation (i.e. by removing from the set of estimated surrogate variables those that are associated with biological confounders) and are subsequently included in our reference-free approach. However, it is somewhat unclear how to proceed when batches are confounded with phenotypes; more detailed research is needed to develop methods that adjust simultaneously for linear mixing effects and non-linear technical effects, using datasets having fully annotated technical data such as chip number and position. However, we view this article as a concrete step in that direction, and our data samples demonstrate the practicality of our method.

Our approach now offers the possibility of conducting EWAS analysis adjusting for mediation by cell type even without the existence of reference datasets that may be expensive or infeasible to collect. If widely adopted, it could pave the way for EWA studies that are more robust with higher potential for replication of results.

## Supplementary Material

Supplementary Data
