# Supplementary material for: Reference-free cell mixture adjustments in analysis of DNA methylation data
Source: Bioinformatics. 2014 Jan 21;30(10):1431–9. doi: 10.1093/bioinformatics/btu029 (PMC4016702; doi:10.1093/bioinformatics/btu029)
Supplement: Supplementary Data [file supp_30_10_1431__index.html]

Reference-Free Cell Mixture Adjustments in Analysis of DNA Methylation Data — Reference-free cell mixture adjustments in analysis of DNA methylation data — Reference-free cell mixture adjustments in analysis of DNA methylation data — Supplementary Data 

# Reference-free cell mixture adjustments in analysis of DNA methylation data

## Supplementary Data

files

**Files in this Data Supplement:**

- Supplementary Data - pdf file
