# Supplementary material for: Reference-free cell mixture adjustments in analysis of DNA methylation data
Source: Bioinformatics. 2014 Jan 21;30(10):1431–9. doi: 10.1093/bioinformatics/btu029 (PMC4016702; doi:10.1093/bioinformatics/btu029)

Reference-Free Cell Mixture Adjustments in Analysis of DNA Methylation Data

E. Andres Houseman[1], John Molitor[1], Carmen J. Marsit[2]
[1]School of Biological and Population Health Sciences, College of Public Health and Human Sciences, Oregon State University, Corvallis, OR 97331, USA
[2]Section of Biostatistics and Epidemiology, Department of Community and Family Medicine, Geisel School of Medicine at Dartmouth, Hanover, NH 03755, USA

Supplementary Information

I. Generation of Intercept and Slope Parameters (B) for Simulation Studies

The 1000×2 matrix $\mathbf{B}$ was generated (once for all simulated data sets) from a 3-part mixture model as follows. Each intercept (first column $\boldsymbol{\beta}_1$ of $\mathbf{B}$) for CpGs assumed not to be differentially methylated regions (DMRs) for different cell types was generated as $\text{logit}^{-1}(a)$, where $a \sim 0.3N(-2.8, 0.7^2) + 0.2N(-0.75, 1.4^2) + 0.5N(1.5, 0.7^2)$. This mixture model was motivated by several HumanMethylation450 data sets, in combination with an overall expectation that only "mid-range" beta values will demonstrate epidemiologically useful covariation with phenotype. See Figure S1(a) for a density plot of the resulting a variables. Note that for DMRs, the implicit intercept was $\mathbf{M}\overline{\boldsymbol{\omega}}$, where $\mathbf{M}$ was the 250×4 matrix representing the methylation profiles for each of 4 cell types and $\overline{\boldsymbol{\omega}}$ was the vector of population average cell proportions. Each slope (second column $\boldsymbol{\beta}_2$ of $\mathbf{B}$) was zero whenever a arose from the first or third component of the 3-part mixture. For variables a arising from the second component, the corresponding slope was generated as $-\text{sgn}(a+0.75)b$, where $b \sim 0.75\,\delta_0 + 0.25\,N(0.4, 0.1)$ and, for the 250 CpGs representing DMRs, $\mathbf{a} := \mathbf{M}\overline{\boldsymbol{\omega}}$. This latter substitution was implemented to ensure that the impact of $\mathbf{B}$ would rarely take the average methylation out of the natural beta scale range, even at DMRs. The scatterplot in Figure S1(b) shows each slope and intercept used in all simulation studies. There were 30 negative slope values, 23 positive slope values, and 947 zero values (i.e. approximately 5% non-null).

Figure S1: Intercept and slope parameters (**B**) for simulation experiments

Panel (a): $\text{logit}(\boldsymbol{\beta}_1)$ for non-DMRs

Panel (b): Scatterplot of $\boldsymbol{\beta}_2$ by $\boldsymbol{\beta}_1$

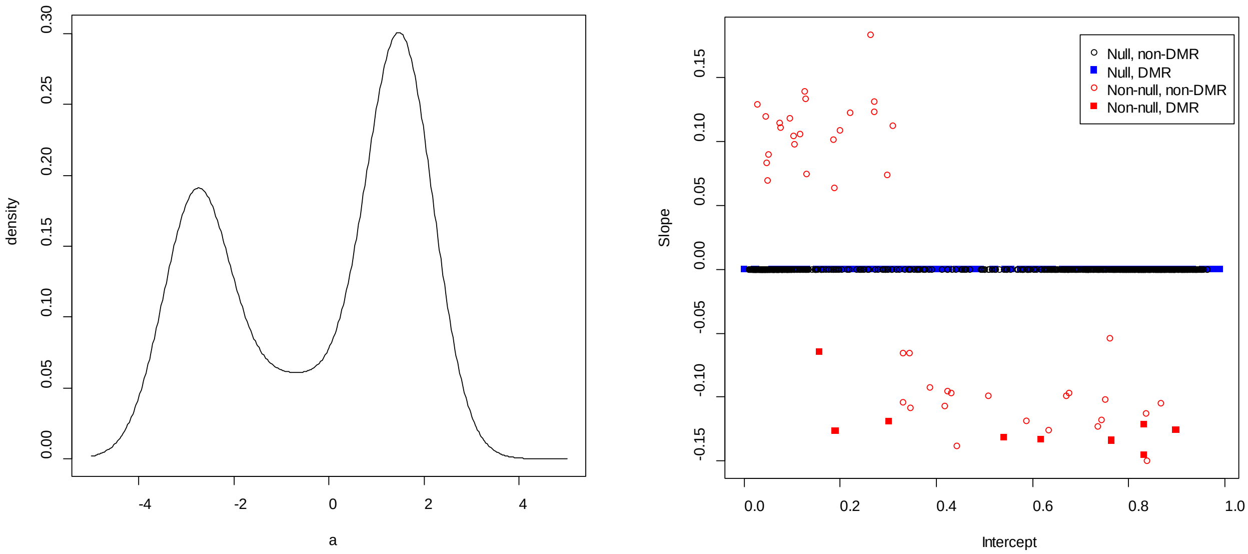

II. Microarray error used in simulations

The box-and-whisker diagrams shown in Figure S2(a) depict the distributions of residual standard deviations for various *limma* regression models applied to M-values (logit-transformed betas), on a common logarithm scale. Each of these models are similar to beta-value models described elsewhere in the main text or in the Supplement, except that M-values were used as outcomes. For example, the "unadjusted" model used for the blood data set (Liu et al., 2013) included only an intercept and a term representing arthritis case/control status, while the "reference-adjusted" model also included terms for leukocyte proportion, a biological covariate, as described in the main text. These distributions represent an upper bound on the technical variation arising from microarray error. The PBMC data set (Lam et al., 2012) admits two biological adjustments, either by monocyte/lymphocyte proportion or by terms representing 5 leukocyte subsets (as described below in Section VII). Note that for the placenta data set (Bannister et al, 2011) it is unclear whether the surrogate variables used in the SVA-adjusted analysis represent biological, technical variation, or a combination. The horizontal blue lines correspond to standard deviations of 0.25 and 0.5. In every analysis, over 75% (actually 95% to 100%) of the CpGs had standard deviations falling below 0.5. Only the unadjusted placenta data analysis yielded a substantial number (>25% of the array CpGs) of standard deviations above 0.25. For all other analyses, more than half of the array CpG standard deviations lay below 0.25, and for PBMCs and SVA-adjusted placenta, close to 75% of the array CpG standard deviations fell below 0.25. Thus, the microarray variation parameters used in the simulation study represent realistic values.

The clustering heatmap shown in Figure S2(b) illustrates a typical simulated data set. Note that this figure is similar to many published in the DNA methylation literature in terms of subtle heterogeneity that drives meaningful but imperfect clustering of CpGs and subjects.

Figure S2: Empirically derived microarray error standard deviations and consequences for simulated data sets

Panel (a): Distribution of standard deviations from M-value analyses

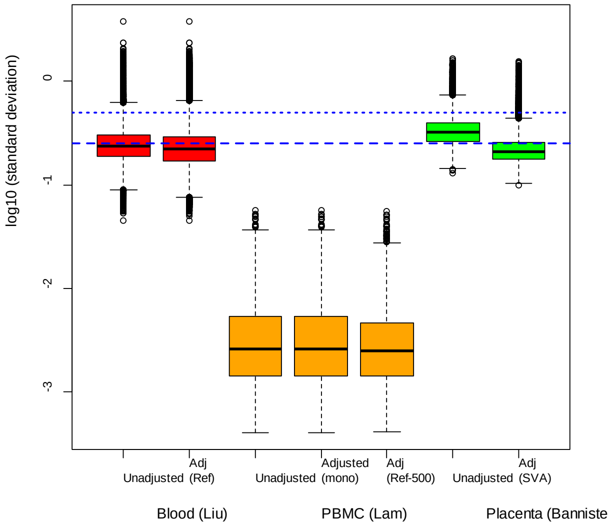

Panel (b): Clustering heatmap depicting typical simulated data set.

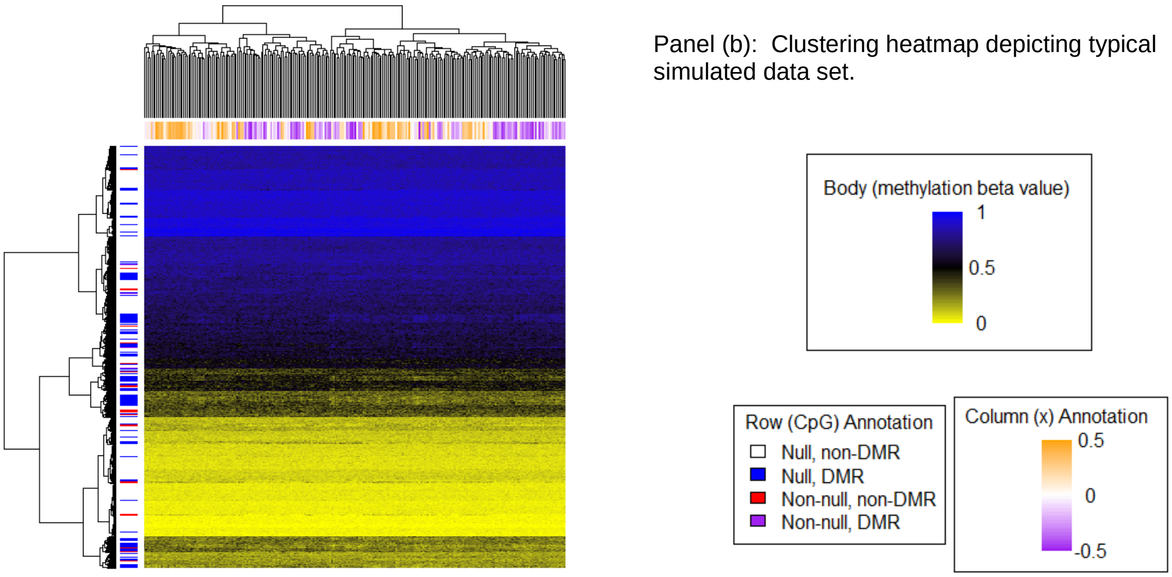

III. Additional details from main simulations

Appearing below are plots similar to Figures 1 and 2 in the main text for simulation scenarios #2, #3, and #4 (described in the main text). In all four simulations, the reference-free method produces estimates that are as good as or superior to SVA and the unadjusted ($\boldsymbol{\beta}_2^*$) methods. In particular, for the null case $\boldsymbol{\beta}_2 = \mathbf{0}$, coefficient estimates at DMRs are much more variable for the SVA and unadjusted methods. For all four simulations, the bootstrap standard error estimation method performs tolerably well, although for the non-null case (Simulations #1 and #2) the method overestimates the standard error slightly, especially at DMRs. For two non-DMR CpGs with non-null slopes, the magnitudes of the standard errors are vastly over-estimated, and for one DMR with zero slope, the standard error is slightly underestimated (thus leading to potential Type I error for that DMR). The two CpGs for which standard error magnitude was overestimated corresponded to intercepts close to the zero boundary, resulting in nonlinear effects for more negative values of the covariate x (due to truncation). We conducted an additional set of simulations using the same parameters as for simulation #1, except that we increased the sample size to n=500. Figure S3(g) and S3(h) compare multiplicative bias for the two sample sizes, suggesting a decrease in bias with the larger sample size, particularly for the two CpGs having extremely biased SE in the smaller-sample case (but also some modest decreases in bias at other CpGs). In particular, among 13 CpGs for which the median estimated SE was over twice the simulation SD with n=250, the median ratio of bias fell 29% from n =250 to n=500. Over all 1000 CpGs, the decrease was 3.5%.

Figure S3: Mean estimates and median standard errors for simulations #2, #3 and #4

Panel (a): Simulation 2 ($\boldsymbol{\beta}_2 \neq \mathbf{0}, \boldsymbol{\gamma}_2^{\mathrm{T}} = \mathbf{0}$): estimates by true parameters (reference-free, SVA, and unadjusted methods)

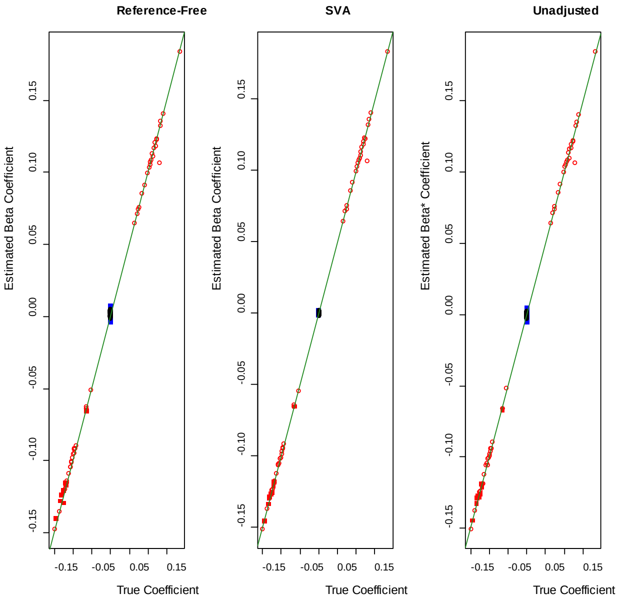

Panel (b): Simulation 2 ($\boldsymbol{\beta}_2 \neq \mathbf{0}, \boldsymbol{\gamma}_2^{\mathrm{T}} = \mathbf{0}$): simulation standard deviation by median standard error (reference-free method)

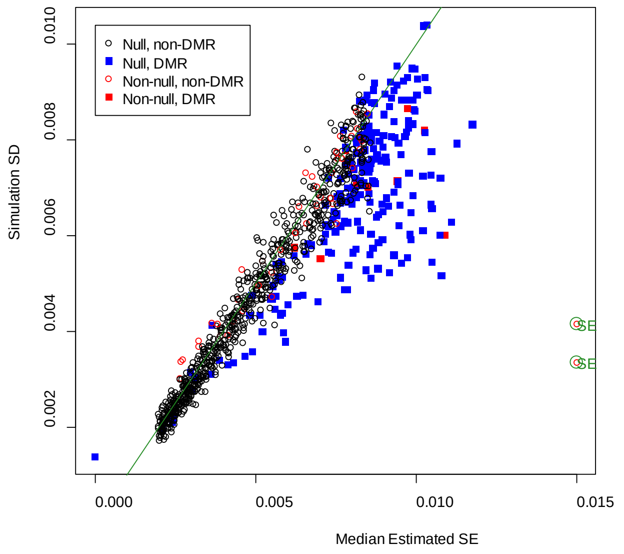

Panel (c): Simulation 3 ($\boldsymbol{\beta}_2 = \mathbf{0}, \boldsymbol{\gamma}_2^{\mathrm{T}} \neq \mathbf{0}$): distribution of estimates (reference-free, SVA, and unadjusted methods)

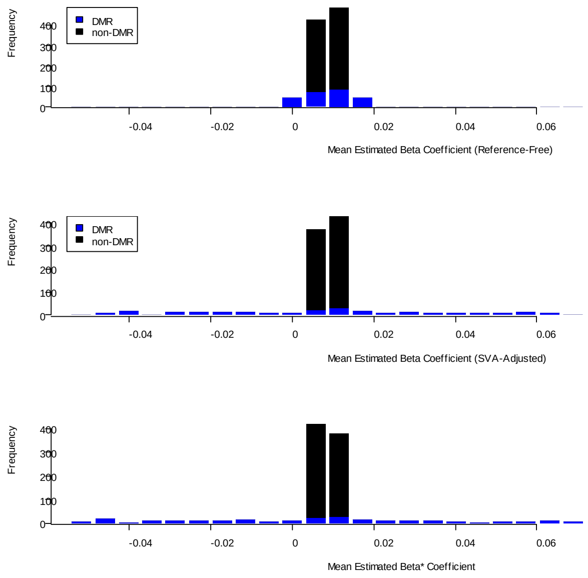

Panel (d): Simulation 3 ($\boldsymbol{\beta}_2 = \mathbf{0}, \boldsymbol{\gamma}_2^{\mathrm{T}} \neq \mathbf{0}$): simulation standard deviation by median standard error (reference-free method)

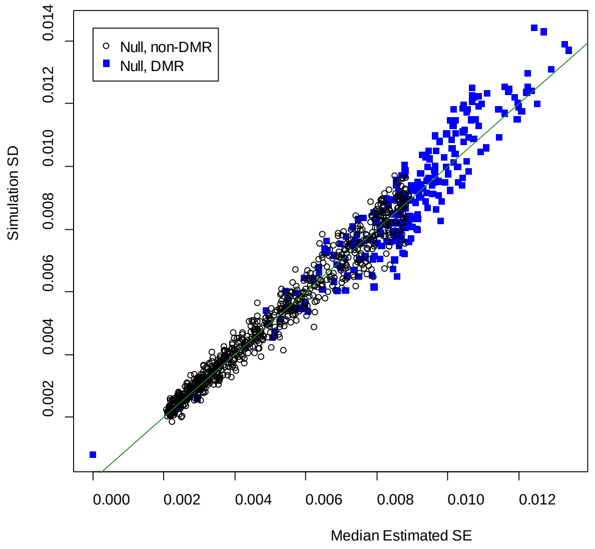

Panel (e): Simulation 3 ($\boldsymbol{\beta}_2 = \mathbf{0}, \boldsymbol{\gamma}_2^T = \mathbf{0}$): distribution of estimates (reference-free, SVA, and unadjusted methods)

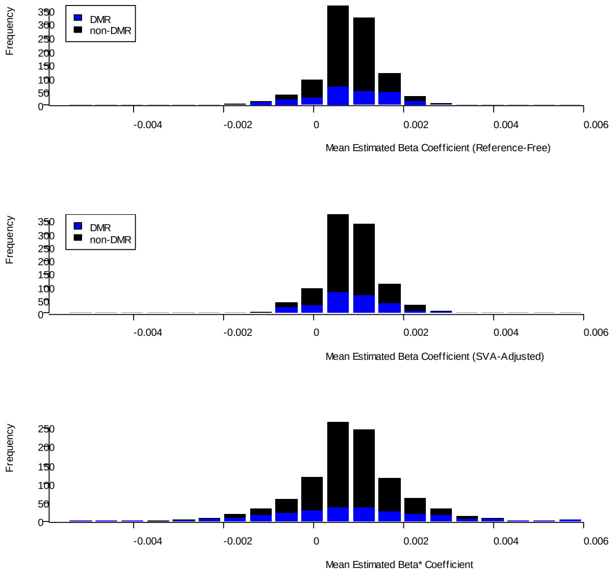

Panel (f): Simulation 3 ($\boldsymbol{\beta}_2 = \mathbf{0}, \boldsymbol{\gamma}_2^T = \mathbf{0}$): simulation standard deviation by median standard error (reference-free method)

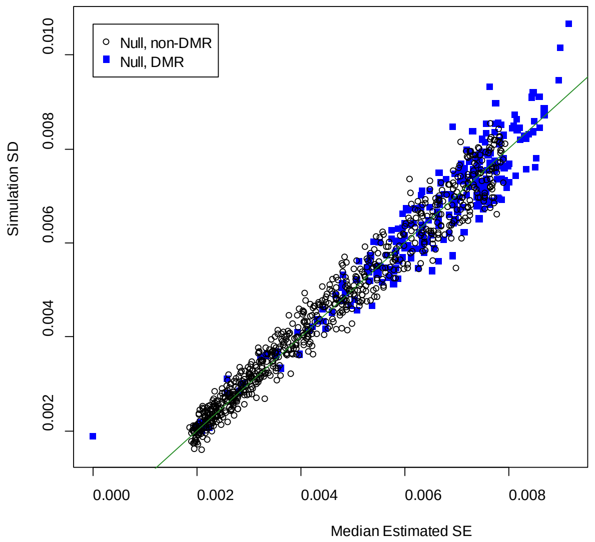

Panel (g): Comparison of multiplicative SE bias between *n*=250 and *n*=500, based on Simulation 1 ($\boldsymbol{\beta}_2 \neq \mathbf{0}, \boldsymbol{\gamma}_2^T \neq \mathbf{0}$)

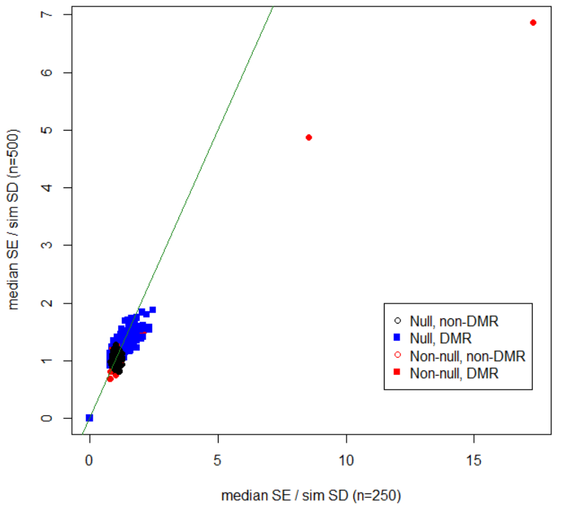

Panel (h): Close-up view of panel (g)

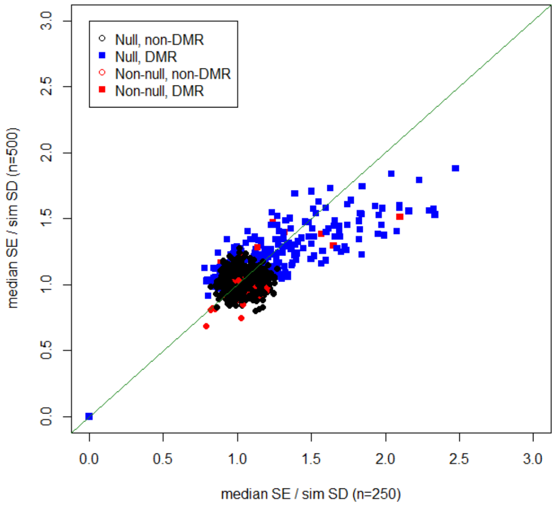

IV. Additional simulations demonstrating the impact of differing microarray variance parameters

We conducted additional simulations in order to investigate the impact of the magnitude of variation and the magnitude of correlation within the microarray error component on dimension estimation and relative error of the proposed reference-free method compared against the SVA method. We started with parameters used in main simulation #1, which posed a standard deviation parameter $\theta = 0.008$ on the beta values representing biological error, and posed a factor analytic structure $\mathbf{\Lambda}_{micro}\mathbf{\Delta}_{micro}\mathbf{U}^{\mathrm{T}}_{micro,i} + \lambda_{micro}\mathbf{E}_{micro,i}$ on the microarray error: $\mathbf{\Delta}_{micro} = \mathrm{diag}(0.25, 0.01)$ diagonal, the elements of $\mathbf{\Lambda}_{micro}$ generated as standard normal variables once for the data set, and the elements of $\mathbf{U}^{\mathrm{T}}_{micro,i}$ and $\mathbf{E}_{micro,i}$ generated as standard normal variables for each individual subject *i*. Note that for each CpG and each subject, the microarray error had variance equal (on average over all CpGs) to $\sigma^2_{micro} := \mathrm{trace}(\mathbf{\Delta}^2_{micro}) + \lambda^2_{micro} = 0.25$, with communal portion equal (on average) to $\mathrm{trace}(\mathbf{\Delta}^2_{micro})$ and the uniqueness portion equal to λ^2_{micro}.

We conducted 24 additional simulation studies, defined by beta variation $\upsilon_1\theta$ and microarray structure having total variance (on average) equal to $(\upsilon_2\sigma_{micro})^2$, with $\upsilon_1 \in \{1,2\}$, $\upsilon_2 \in \{1,2\}$, and having factor analytic structure $\upsilon_3\mathbf{\Lambda}_{micro}\mathbf{\Delta}_{micro}\mathbf{U}^{\mathrm{T}}_{micro,i} + [(\upsilon_2\sigma_{micro})^2 - \upsilon_3^2\,\mathrm{trace}(\mathbf{\Delta}^2_{micro})]\mathbf{E}_{micro,i}$, with υ_3 chosen so that the communal portion $\upsilon_3^2\,\mathrm{trace}(\mathbf{\Delta}^2_{micro})/(\upsilon_2\sigma_{micro})^2 \in \{0.01, 0.5, 0.6, 0.75, 0.9, 0.99\}$.

The point of these parameterization choices is to demonstrate variation in magnitude of correlation across two different scales of overall microarray variation and over two different levels of biological dispersion. In addition to evaluating RMSE, we wanted to compare the RMT method of dimension estimation proposed by of Teschendorff et al. (2012) to simpler alternatives based on minimizing AIC and BIC over candidate dimensions d'. These information criteria were based on normal log-likelihoods assuming an independence model across CpGs and subjects, computed as follows. After computing the SVD of the (unadjusted) residual matrix $\hat{\mathbf{E}}^*$, we consider a candidate latent variable dimension d' and use the first d' dimensions of the decomposition (the ones corresponding to the largest d' singular values) to compute candidate error matrix $\mathbf{E}'_{d'} = \hat{\mathbf{E}}^* - \mathbf{L}'_{d'}\mathbf{\Delta}'_{d'}\mathbf{U}'_{d'}$, where $\mathbf{L}'_{d'}$ is the appropriate $m \times d'$ orthogonal matrix, $\mathbf{\Delta}'_{d'}$ is the appropriate $d' \times d'$ diagonal matrix, and $\mathbf{U}'_{d'}$ is the appropriate $d' \times n$ orthogonal matrix. Under the model (i.e. d' chosen appropriately), $\mathbf{E}'_{d'}$ has independent rows and columns. Letting $\hat{\sigma}^2_j(d') = n^{-1}\sum_{i=1}^{n} e'^2_{d',ij}$ be the estimated variance of row j of $\mathbf{E}'_{d'}$ (computed with n degrees of freedom, as the mean is known to be zero), the log-likelihood for each row is $-n\{1+\log[\hat{\sigma}^2_j(d')]\}/2$, and thus the total log-likelihood under independence is $\ell_{d'} = -\sum_{j=1}^{m} n\{1+\log[\hat{\sigma}^2_j(d')]\}/2 = -\frac{1}{2}\{mn + n\sum_{j=1}^{m}\log[\hat{\sigma}^2_j(d')]\}$. Since there are $d'(m+n)$ unique parameters involved in the construction $\mathbf{L}'_{d'}\mathbf{\Delta}'_{d'}\mathbf{U}'_{d'}$ (due to scale non-identifiability, the diagonal of $\mathbf{\Delta}'_{d'}$ does not count) and m distinct $\hat{\sigma}^2_j$ quantities, the number of free parameters is $m + d'(m+n)$; thus AIC is computed as $2[m + d'(m+n)] - 2\ell_{d'}$ and BIC is computed as $\log(n)[m + d'(m+n)] - 2\ell_{d'}$.

Figure S4(a) shows average estimated dimension by AIC, BIC, and the RMT method of Teschendorff et al. (2012) across six levels of communal variation for $\upsilon_1 = \upsilon_2 = 1$, while Figure S4(b) shows average estimated dimension for the RMT method over all communalities and all four choices of (υ_1, υ_2). In both panels, the horizontal red line indicates the true dimension of the latent structure (3 linearly independent cell proportions and 2 microarray latent variables). The pattern in Figure S4(a) was representative of the other three choices of (υ_1, υ_2) (not shown). It becomes evident that the RMT method was superior to both AIC and BIC, and almost always estimated the correct dimension for larger communality

parameters. For the smallest communality parameter (1% communality) RMT had difficulty estimating the full dimensionality, presumably because the microarray latent dimensions were too faint to detect.

Figures S5(a) and S5(b) show the total RMSE for different dimensions (true dimension, BIC-estimated dimension, and RMT-estimated dimension) and different methods of parameter estimation (unadjusted, reference-free-adjusted, and SVA-adjusted) across the 6 different communality parameters, for $\upsilon_2 = 1$ and $\upsilon_2 = 2$ respectively (with $\upsilon_1 = 1$ in both cases). The plots suggest that using the dimension estimated by RMT produces results as good as those obtained using the true dimension, while the use of BIC results in inflated RMSE (presumably because of dimension estimates that are too small, missing important sources of communal variation). Note that smaller communality parameters result in greater RMSE, presumably because the greater level of independent variation leads to less precise estimation. Figures S5(c) and S5(d) show the corresponding CpG-specific RMSE values for reference-free and SVA methods using dimensions estimated by RMT. They also suggest that when the total microarray variation $(\upsilon_2 \sigma_{micro})^2$ is large, the reference-free and SVA methods have about the same level of error (or the reference-free method produces slightly greater error), presumably because the microarray error swamps the biological error. However, when the microarray error is smaller, and its communal proportion is substantial, the reference-free method outperforms SVA, particular in estimating coefficients for DMRs, presumably because the cell-mixture property is explicitly used in supervising the deconvolution. Figure S5(e) summarizes the total RMSE for all four choices of (υ_1, υ_2) at two mid-range levels of communality, based on RMT-estimated dimension; this figure reinforces the superiority of the reference-free approach for mid-range communalities and smaller levels of microarray error.

Figure S4: Simulations results for estimation of dimension

Panel (a) Mean estimated dimension for $\upsilon_1 = \upsilon_2 = 1$

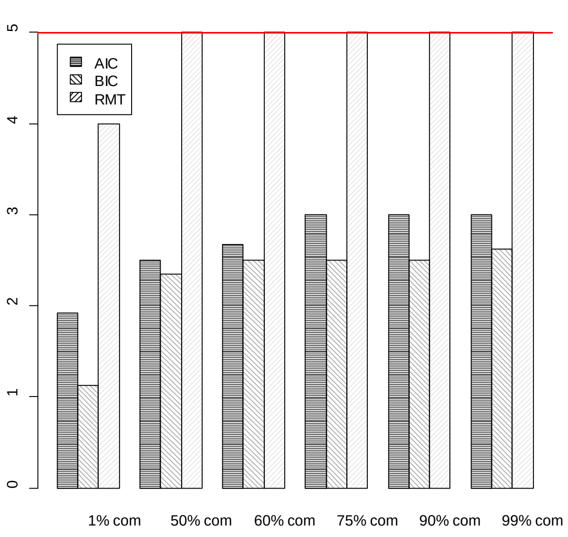

Panel (b) Mean estimated dimension for the RMT method

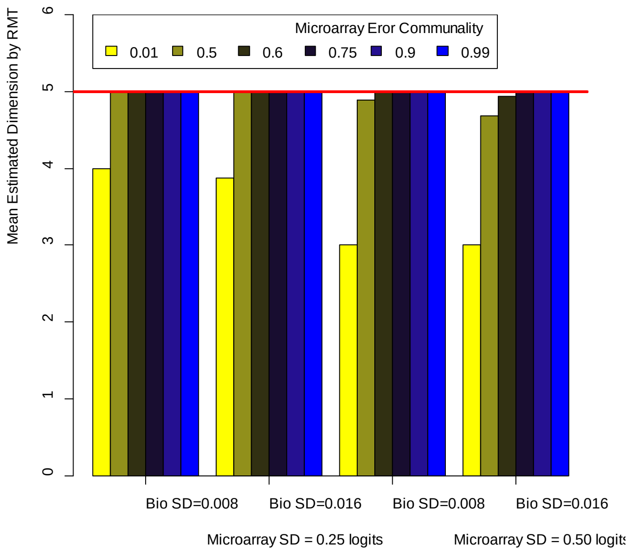

Figure S5: Simulations results for estimation of slope parameters

Panel (a) Total RMSE for $\upsilon_1 = \upsilon_2 = 1$, for various dimension and coefficient estimation methods and various communalities

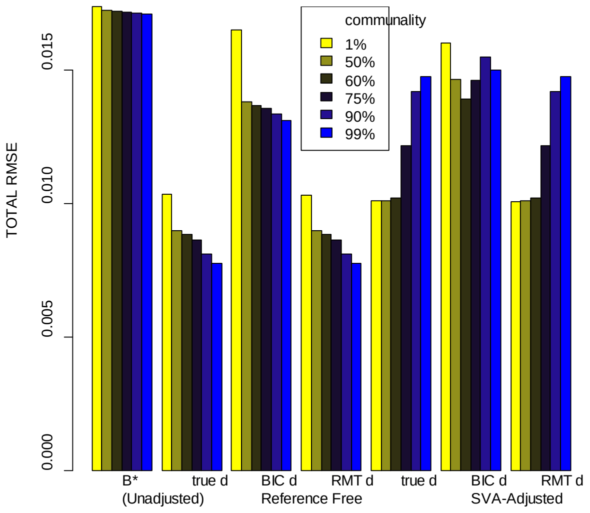

Panel (a) Total RMSE for $\upsilon_1 = 1, \upsilon_2 = 2$, for various dimension and coefficient estimation methods and various communalities

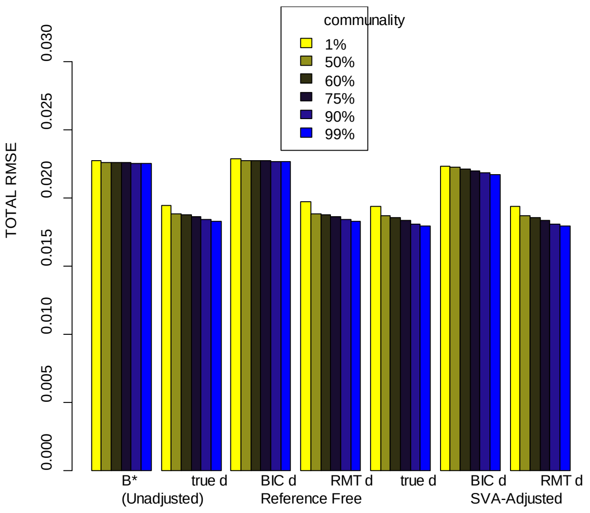

Panel (c) CpG-specific RMSE for $\upsilon_1 = \upsilon_2 = 1$, various coefficient estimation methods, various communalities, and RMT dimension estimation

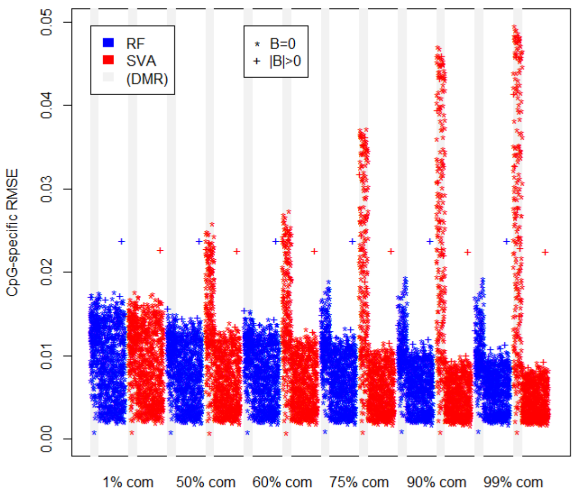

Panel (d) CpG-specific RMSE for $\upsilon_1 = 1, \upsilon_2 = 2$, various coefficient estimation methods, various communalities, and RMT dimension estimation

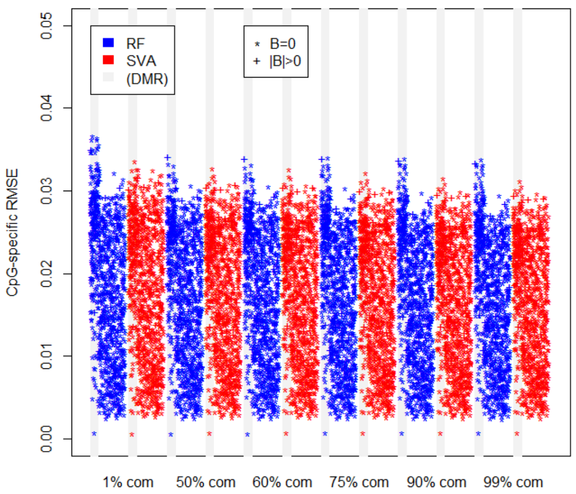

Panel (e) Total RMSE for all four combinations of (υ_1, υ_2) , various coefficient estimation methods, two mid-range communalities, and RMT dimension estimation

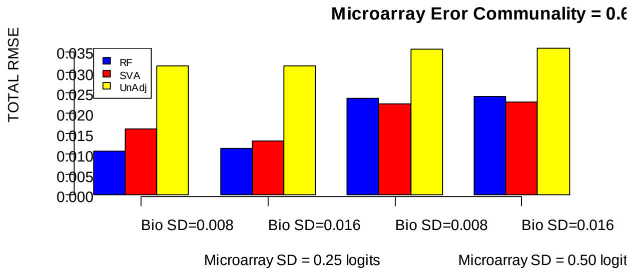

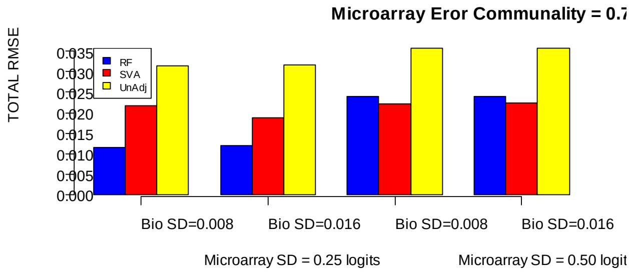

V. Additional simulations illustrating power and Type I error for omnibus significance testing

We first propose a simple method of omnibus significance testing using the bootstrap sampling procedure proposed in the main text, then describe a simulation experiment used to test the approach.

The proposed bootstrap procedure is designed to retain the correlation structure across CpGs. Consequently, if the bootstrap samples can be used to generate an approximate distribution for the individual, CpG-specific null case, then an omnibus test of $\boldsymbol{\beta}_2 = \mathbf{0}$ is possible. Let $(\hat{\boldsymbol{\beta}}_2^{(1)}, \hat{\boldsymbol{\beta}}_2^{(2)}, ..., \hat{\boldsymbol{\beta}}_2^{(R)})$ be bootstrap estimates of the slope $\boldsymbol{\beta}_2$. For each bootstrap sample *r*, let $\boldsymbol{\Sigma}_{boot}^{-1/2}\left(\hat{\boldsymbol{\beta}}_2^{(r)} - \bar{\hat{\boldsymbol{\beta}}}_2^{(\bullet)}\right)$ be the vector of (presumably null) t-statistics computed by centering the bootstraps at the bootstrap mean $\bar{\hat{\boldsymbol{\beta}}}_2^{(\bullet)} = R^{-1}\sum_{s=1}^{R} \hat{\boldsymbol{\beta}}_2^{(s)}$ and rescaling by the diagonal bootstrap variance matrix $\boldsymbol{\Sigma}_{boot}$ of element-wise bootstrap variances $\boldsymbol{\Sigma}_{boot} = \mathrm{diag}\left[(R-1)^{-1}\sum_{s=1}^{R}(\hat{\boldsymbol{\beta}}_2^{(s)} - \bar{\hat{\boldsymbol{\beta}}}_2^{(\bullet)})^2\right]$, and for an individual bootstrap *r* let $(p_{(1)}^{(r)}, p_{(2)}^{(r)} ..., p_{(m)}^{(r)})$ be the *order statistics* across the $m \times 1$ array of the corresponding *p*-values computed as tail probabilities from the a t-distribution with $n-p$ degrees of freedom ($250-2$ in the case of the simulation). Note that these *p*-values will be marginally uniform but correlated across CpGs. The Kolmogorov statistic $\kappa_r = \max_j \{|\, p_{(j)}^{(r)} - (j-0.5)/m \,|\}$ therefore measures deviation from null (uniform) distribution of p-values. The observed test statistic is computed as $\kappa_0 = \max_j \{|\, p_{(j)}^{(0)} - (j-0.5)/m \,|\}$, where $(p_{(1)}^{(0)}, p_{(2)}^{(0)} ..., p_{(m)}^{(0)})$ are the order statistics of the p-values computed as tail probabilities from a t-distribution with $m-p$ degrees of freedom, using observed t-statistics $\boldsymbol{\Sigma}_{boot}^{-1/2}\hat{\boldsymbol{\beta}}_2$. The omnibus p-value is thus computed as $R^{-1}\sum_{r=1}^{R} 1(\kappa_0 \le \kappa_r)$. Note that a similar approach can be used to assess the significance of the unadjusted coefficients, $\mathbf{B}^*$.

Our simulation design was similar to that of main simulation #1, except that the effect of β_2 was assumed to be applied on the M-value (logit) scale rather than the additive beta-value scale. The reason for this change was that in order to investigate the impact of “large” effects, it was necessary to apply the direct-effect coefficients on a logit scale to avoid numerous boundary violations of the mean effect. Note that this choice leads to a multiplicative bias in estimating β_2; by the delta-method (Taylor series approximation) it can be shown that this bias should be multiplicative by a factor of 0.25. For each of 6 choices of multipliers (0,1,2,3,4,5) on the β_2 value used in Simulation #1, we constructed 100 simulated data sets, and to each such data set we applied our proposed omnibus test. Figure S6(a) shows the resulting quantile-quantile (QQ)-plots of omnibus *p*-values (obtained from analyses that used dimension estimated via RMT), superimposed over a region corresponding to the 95% confidence band for QQ-plots of samples from a uniform distribution with sample size 100. The figure suggests that the null case ($\beta_2 = 0$) fits comfortably within the null region, while the other choices lie outside the null region, and demonstrating increasing deviation from uniform as the magnitude of β_2 increases. Figure S6(b) shows true vs. estimated slope parameters for the largest magnitude (5x) case. The anticipated multiplicative bias of 0.25 is evident, along with some additional additive bias due to discrepancies at the low and high ends of the beta-value scale. Still, it is clear from the simulations that nonzero effects are detectable with reasonable power, and that Type-I errors are tolerably close to a uniform distribution. Figures S6(c) through S6(f) demonstrate the impact of effect size on various quantities arising from the application of q-value methodology (via the R package *qvalue*); these are consistent with S6(a).

Figure S6: Simulations results for omnibus p-values

Panel (a): Quantile-quantile plots of omnibus p-values

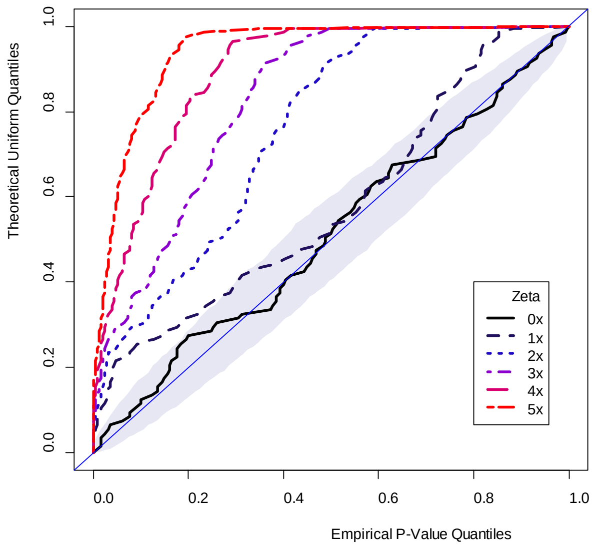

Panel (b) Estimated vs. true slopes for largest magnitude case

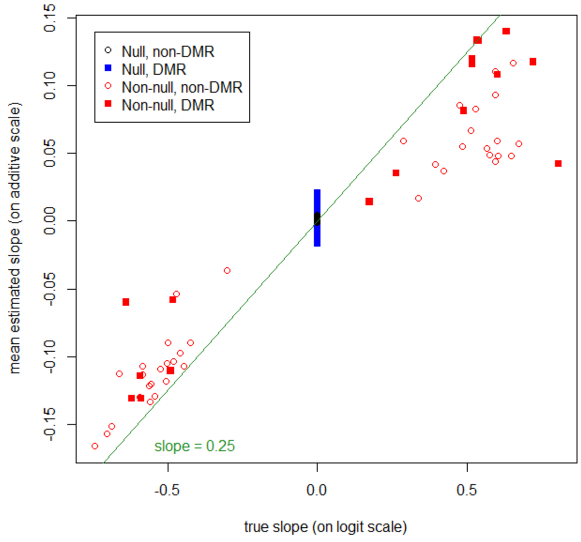

Panel (c): Estimated proportion of null CpGs via *qvalue*

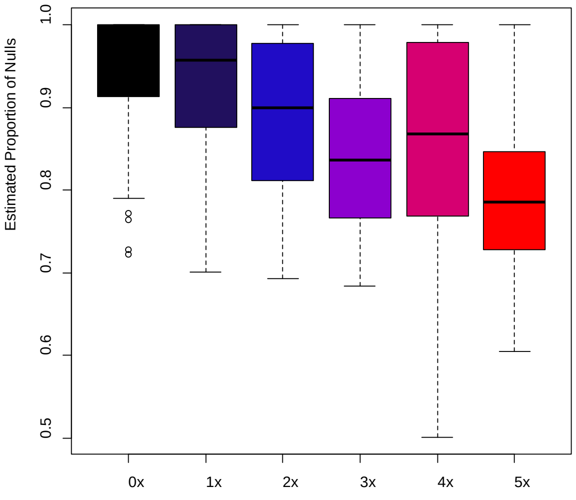

Panel (c): Minimum q-value

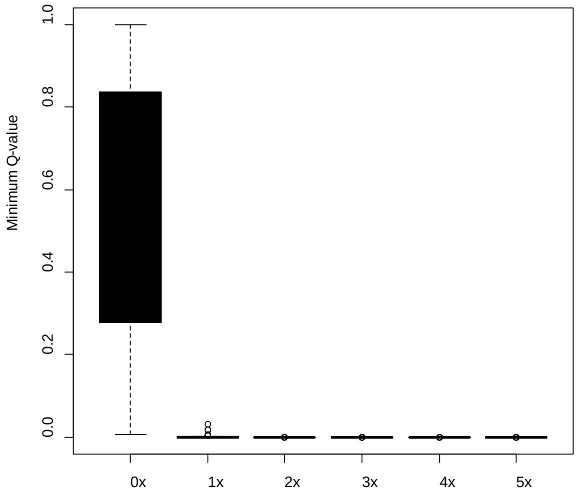

Panel (e): Proportion of CpGs with q<0.05

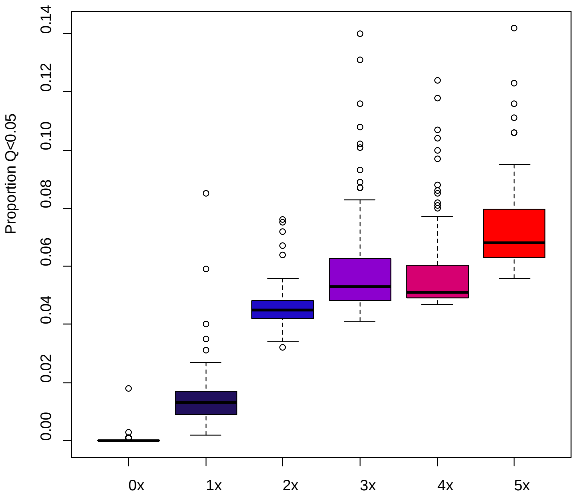

Panel (f): Proportion of CpGs with q<0.10

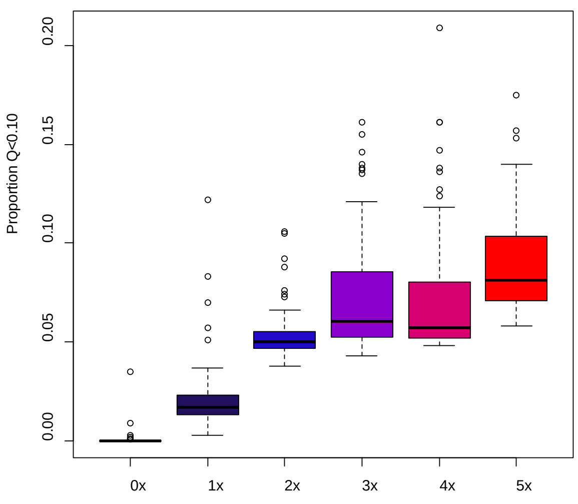

VI. Additional details for analysis of blood/arthritis data set (Liu et al., 2013)

Comparing each of several methods (reference-free vs. unadjusted, reference-free vs. reference-adjusted, and reference-free vs. SVA), Figure S7 shows scatter plots of arthritis coefficient estimates and standard errors. Figure S8 shows the corresponding comparison of significance ($-\log_{10}$ p-values) as well as a volcano plot for SVA-adjusted, reference-adjusted, and reference-free methods. Most of these plots identify in red the 387 values from CpGs in the reference set supplied by Houseman et al. (2012) and overlapping with the Illumina Infinium HumanMethylation450 array, "known DMRs". Finally, the bar plot in Figure S9 shows RMSE values for reference-free analysis and SVA-adjusted analysis with d=37 and d=53, where the assumed gold standard was the analysis based on the reference- adjusted method.

From these figures, it is evident that the unadjusted analysis produced standard errors that were smaller than those produced by the reference-free approach at many non-DMRs, but larger for CpGs known to be DMRs as well as a great many other CpGs [Figure S7(b)]. For 79.7% of the CpGs, the unadjusted analysis produced standard errors that were greater than those produced by the reference-free analysis; nevertheless, significance was vastly increased in the unadjusted analysis compared with the reference-free method, indicating much larger effect sizes. Indeed, many coefficients that were close to zero by the reference-free method had much larger magnitude in the unadjusted analysis, especially for known DMRs [Figure S7(a)]. Interestingly, the SVA analysis produced patterns similar to the unadjusted analysis [Figure S7(e)] in comparison to reference-free [Figure S7(a), comparison of Figure S8(d) with main text Figure 3, and comparison of Figure S8(a) with Figure S8(d)], but with a lesser degree of discrepancy; this was true even though the SVA standard errors were larger than those produced by the reference-free method only at 1.6% of the CpGs [see Figure S7(f)]. The reference-based and reference-free methods produced estimates and standard errors that were much more similar, although it is evident that there were a set of CpGs that had noticeably smaller standard errors in the reference-free method [Figure S7(d)], and reference-based standard errors were larger than reference-free standard errors at 95.2% of the CpGs); in addition, a group of effect estimates had noticeably smaller magnitude in the reference-free approach [Figure S7(c)]. Finally, the standard errors from the reference-based analysis were much smaller than those from the unadjusted analysis at known DMRs [Figure S7(h)], but larger overall, with 87.7% of the CpGs displaying larger standard errors for the reference-adjusted analysis compared with the reference-free. The diminished magnitude at some CpGs in the reference-free approach may account for the apparent decreased significance in the reference-free approach relative to the reference -based approach, and may suggest the existence of cell types that were not profiled (e.g. Tregs and helper-T cells) but that nevertheless drive some of the phenotypic differences. Note that the SVA approaches produce slightly larger total RMSE, compared with the reference-free approach (Figure S9). Finally, the omnibus significance test described in Section V of this Supplement produced p<0.002 for both the unadjusted analysis ($\boldsymbol{\beta}_2^*$) and for the reference-free adjusted analysis ($\boldsymbol{\beta}_2$), indicating omnibus significance that holds up even after the seemingly extreme effect of cell-mixture adjustment.

Figure S7: Comparisons of estimates and standard errors for blood/arthritis data set (Liu et al., 2013)

(a) Panel (a): Arthritis coefficient estimates, reference-free (*d*=37) vs. unadjusted

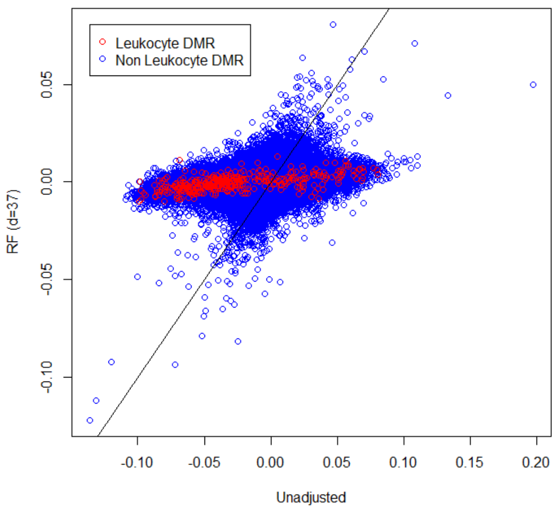

Panel (b): Arthritis coefficient standard errors, reference-free (*d*=37) vs. unadjusted

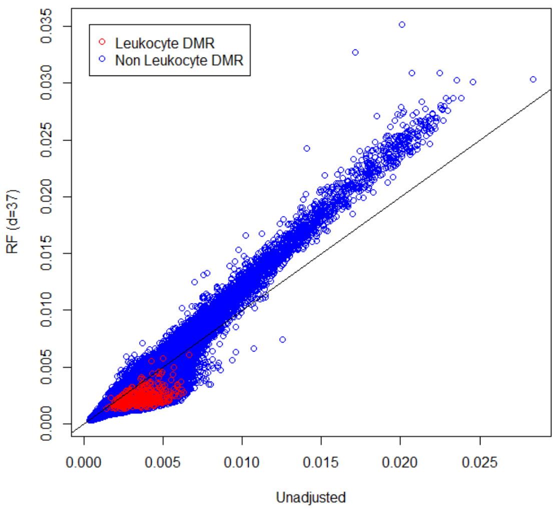

Panel (c): Arthritis coefficient estimates, reference-free (*d*=37) vs. reference-adjusted

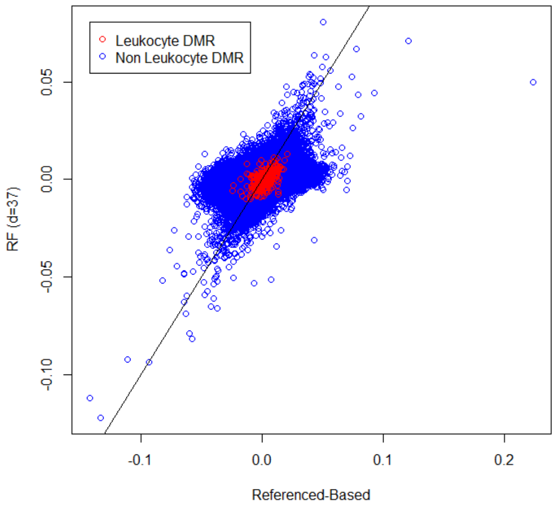

Panel (d): Arthritis coefficient standard errors, reference-free (*d*=37) vs. reference-adjusted

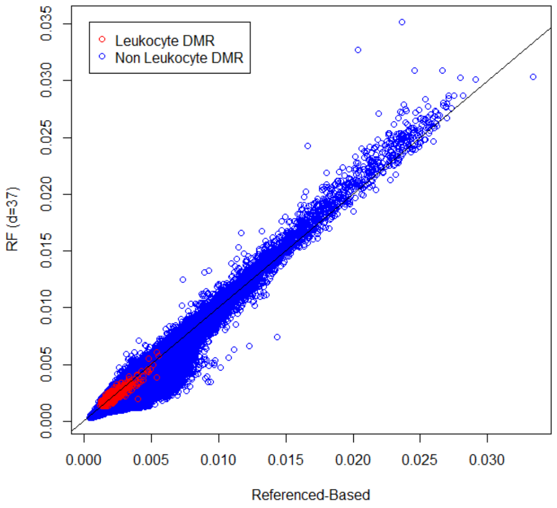

Panel (e): Arthritis coefficient estimates, reference-free (*d*=37) vs. SVA-adjusted (*d*=53)

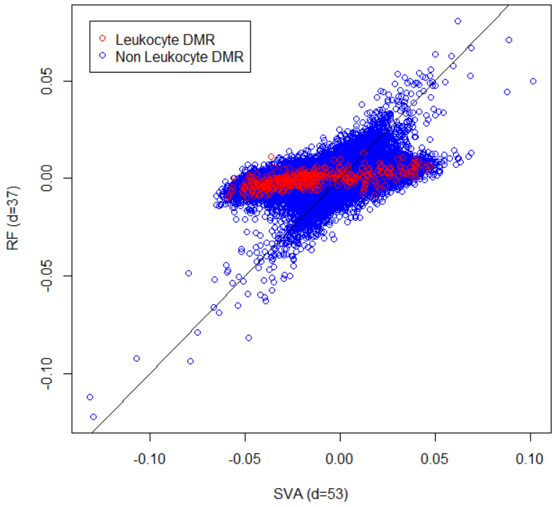

Panel (f): Arthritis coefficient standard errors, reference-free (*d*=37) vs. SVA-adjusted

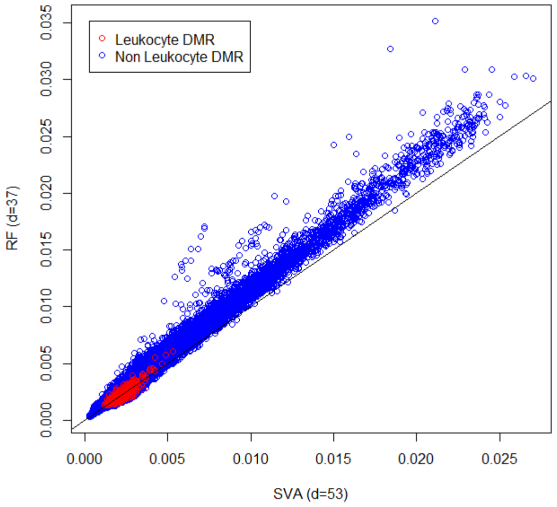

Panel (g): Arthritis coefficient estimates, reference-adjusted vs. unadjusted

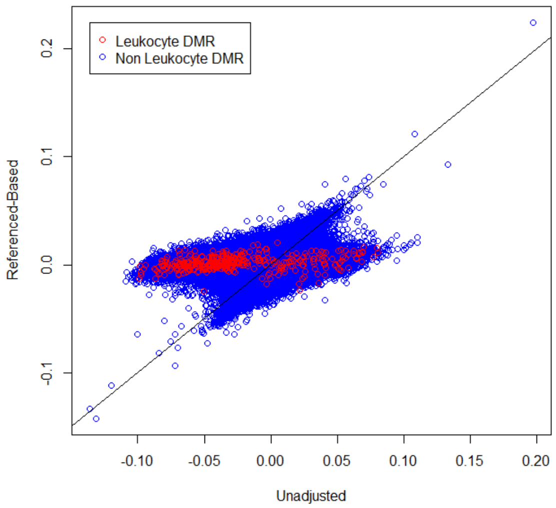

Panel (h): Arthritis coefficient standard errors, reference-adjusted vs. unadjusted

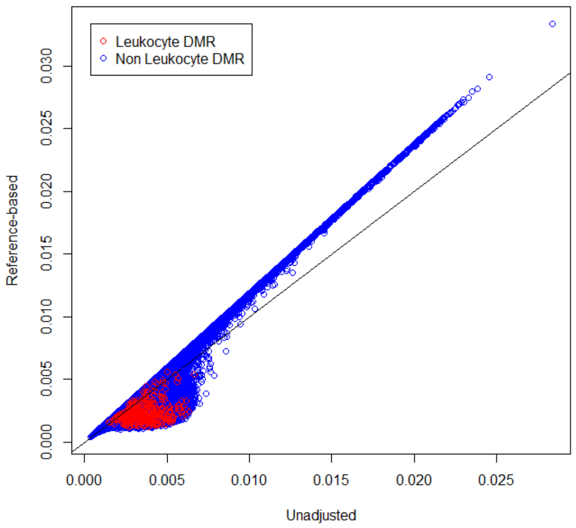

Figure S8: Comparisons of significance for blood/arthritis data set (Liu et al., 2013)

Panel (a): Negative $\log_{10}$ p-values, reference-free (*d*=37) vs. unadjusted

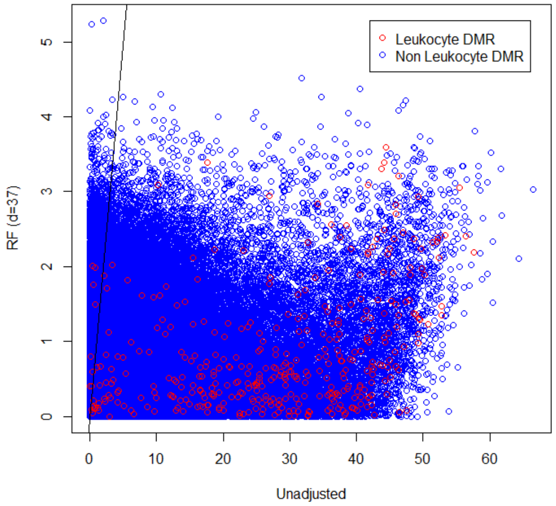

Panel (b): Negative $\log_{10}$ p-values, reference-free (*d*=37) vs. reference-adjusted

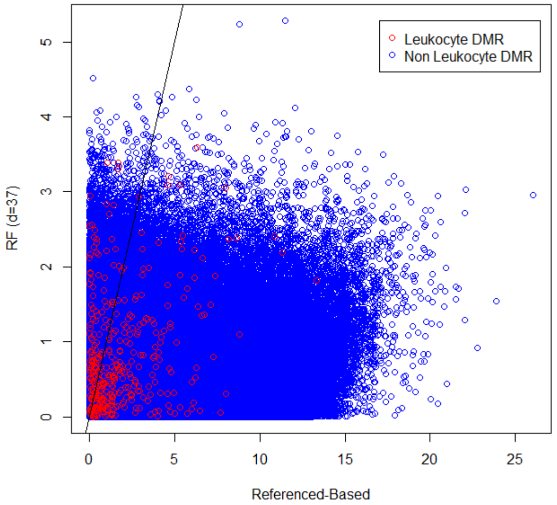

Panel (c): Negative $\log_{10}$ p-values, reference-free (*d*=37) vs. SVA adjusted (*d*=53)

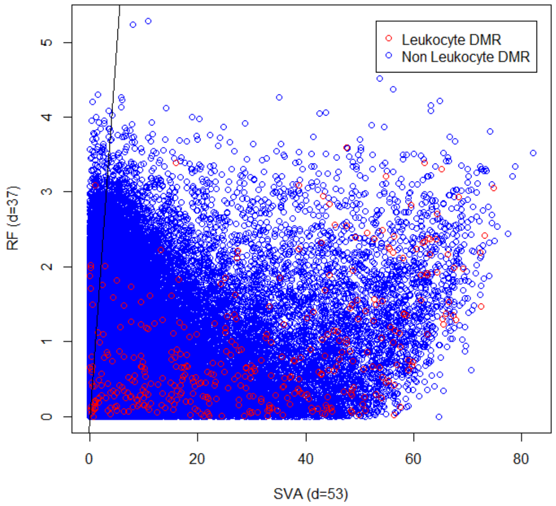

Panel (d): Additional volcano plots

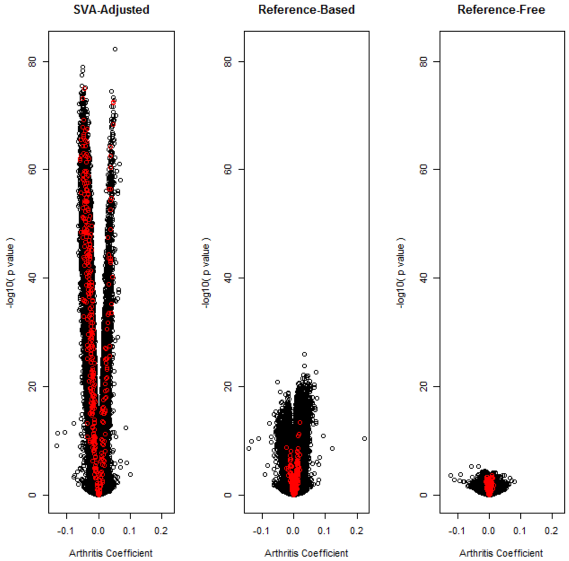

Leukocyte DMRs indicated in red

Figure S9: Comparison of total RMSE for different analyses of blood/arthritis (Liu et al., 2013) data set

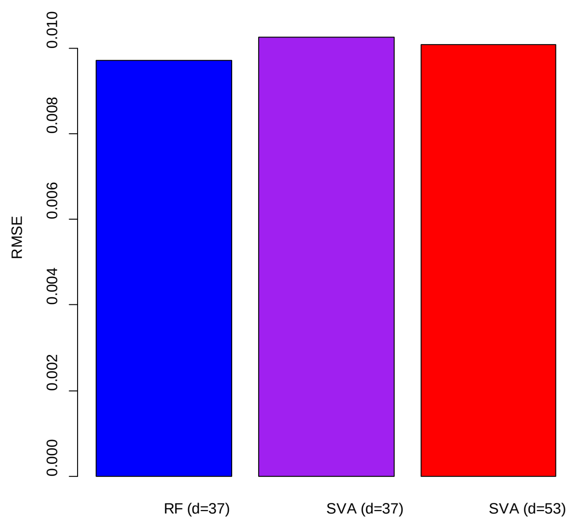

VII. Analysis of PBMC data set (Lam et al., 2012)

Our second analysis consisted of Illumina Infinium HumanMethylation27array data for 92 independent peripheral blood mononuclear cell (PBMC) samples, originally published in Lam et al. (2012) and available in GEO, Accession number GSE37008. Note that, for the purposes of this analysis, PBMC samples can be thought of as whole blood with granulocytes removed. In addition to DNA methylation data, Complete Blood Count (CBC) differential data were available for each sample, thus providing gold standard estimates for the fraction of the PBMC sample consisting of monocytes, assumed to be one minus the fraction of lymphocytes (including B, T and NK cells). Using several different approaches applied to the autosomal subset of the array data, we examined the association between DNA methylation and the logarithm of il6 response to phorbol-12-myristate-13-acetatein ("log pma"), a potent promoter of cell division. Note that this variable was associated with both monocyte fraction (-8.3 percentage points per logarithm, 95% confidence interval from -11.2 to -5.4, p<0.0001) and with the six cell proportions profiled in Houseman et al., 2012, the latter assessed using the methods described in Houseman et al., 2012 (p<0.0001). The first analysis was unadjusted; the second analysis was adjusted for monocyte fraction; the third and fourth analyses were adjusted for blood cell fractions estimated using the approach of Houseman et al. (2012), similar to the approach described above, with the top 100 or 500 DMRs published in Houseman et al. (2012). In the fifth approach, we applied the reference-free approach proposed in this article, with $d = 10$. Note that the RMT dimension estimation method of Teschendorff et al. (2011) produced $d = 10$; AIC produced $d = 10$, and BIC produced $d = 6$. We also applied SVA with d=10 and d=11, the latter being the dimension estimated using the "be" option of the R package *sva* (version 3.6.0), i.e. the method implemented in the *num.sv* function and proposed by Buja and Eyuboglu (1992).

Q-values were computed for each of the five approaches using the R package *qvalue*. The first approach produced 503 CpG coefficients having q<0.05 and 603 CpG coefficients having q<0.1; in addition, the *qvalue*-estimated proportion of non-null coefficients was 0.0011, and there were 93 CpGs whose coefficients reached Bonferroni-adjusted significance of p<0.05/26486. None of the other approaches (reference-based adjustments, reference-free adjustment, and SVA adjustments) produced any CpG coefficients having q<0.1. The omnibus significance test described in Section V of this

Supplement produced p=0.77 for the unadjusted analysis ($\boldsymbol{\beta}_2^*$) and p=0.40 for the reference-free adjusted analysis ($\boldsymbol{\beta}_2$). These results suggest that even though there was apparently elevated significance of unadjusted associations between DNA methylation and log pma at some CpGs (notably DMRs for leukocyte proportion), an insufficient proportion of CpGs had significant unadjusted associations to alter the p-value distribution across all CpGs in a way that was significantly distinct from a uniform distribution, and significance vanished entirely after adjusting for cell proportion.

See Figures S10 for graphical results: comparison of volcano plots, comparison of coefficient estimates, comparison of standard errors, and comparison of total RMSE among reference-free and SVA-adjusted analyses (where estimates from either the monocyte-adjusted model or adjustment by proportions based on the 500-DMR reference data set were used as the gold standard). The 500 DMR CpGs supplied by Houseman et al., 2012, are indicated on many of these plots. In general, all adjusted analyses reduced significance relative to the unadjusted analysis in roughly the same amounts, and the adjusted models tended to shrink the coefficient estimates of many CpGs (especially DMRs) relative to the unadjusted model. The reference-free model produced standard errors that were slightly smaller than the reference-based and SVA-adjusted models. The SVA-adjusted models produced standard errors that were slightly larger than the reference-based model. The two reference-based adjustments produced extremely similar, though not identical, results, and the two SVA-based adjustments produced similar, though not identical results. RMSE values for the reference-free method were lower than the corresponding values for the SVA-adjusted methods.

Figure S10: Graphical analysis of results of PBMC data set (Lam et al., 2012)

Panel (a): Volcano Plots (I) Panel (b): Volcano Plots (II)

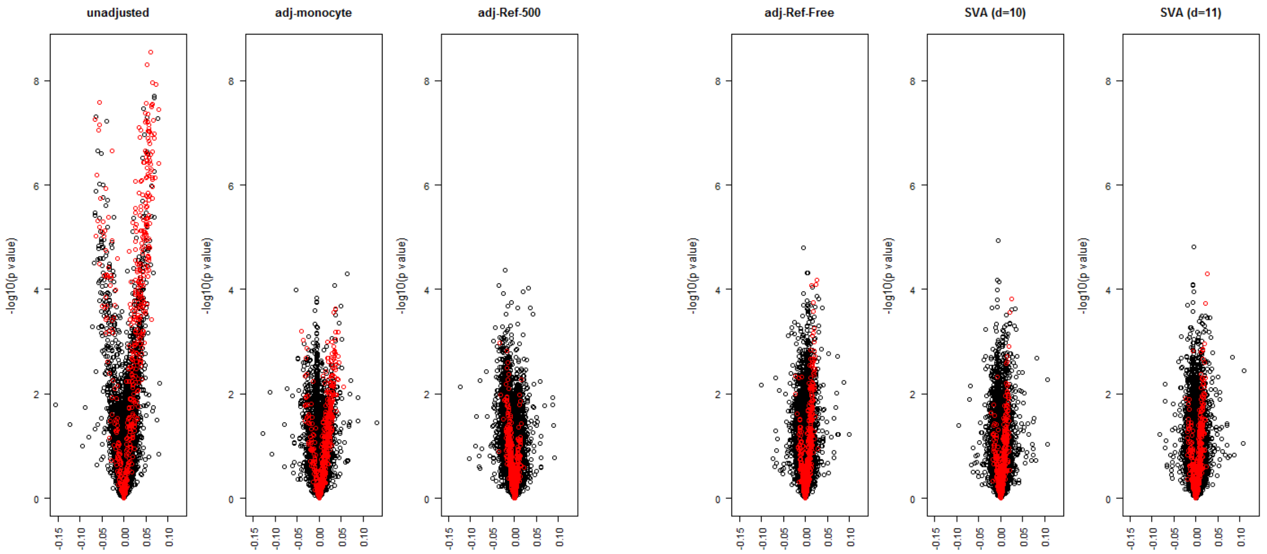

Panel (c): PMA coefficient estimates, reference-free (d=10) vs. unadjusted

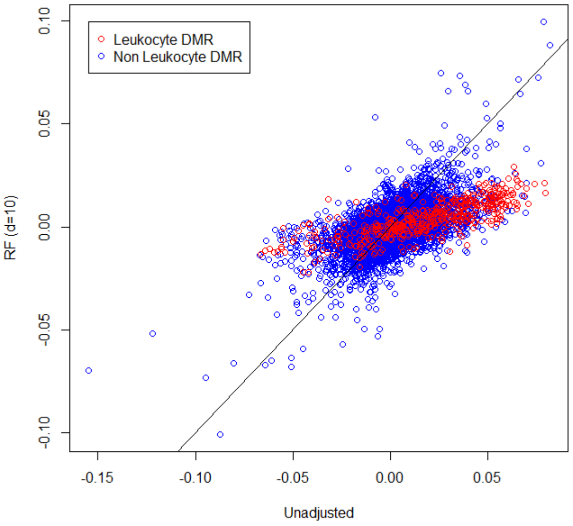

Panel (d): PMA standard errors, reference-free (d=10) vs. unadjusted

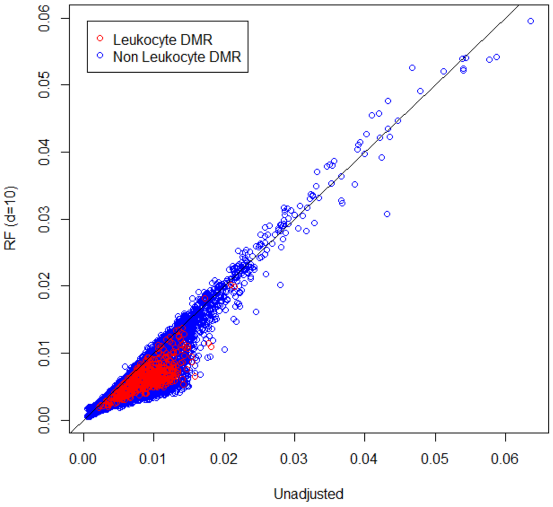

Panel (e): PMA coefficient estimates, reference-free (d=10) vs. reference-adjusted (500 DMRs)

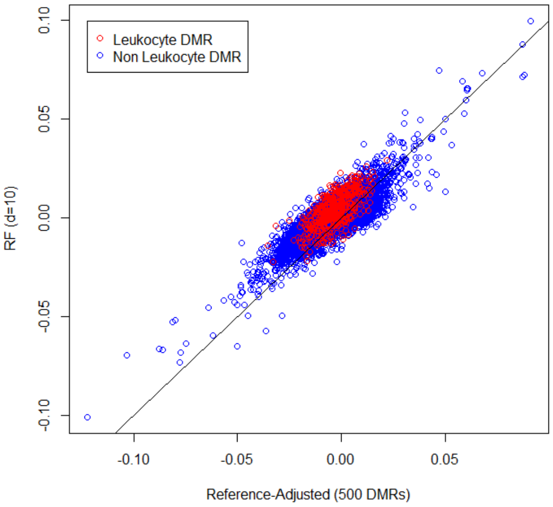

Panel (f): PMA standard errors, reference-free (d=10) vs. reference-adjusted (500 DMRs)

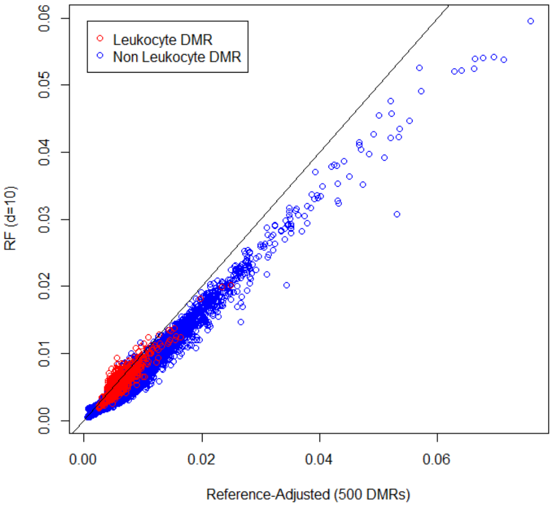

Panel (g): PMA coefficient estimates, reference-free (d=10) vs. SVA (d=11)

Panel (h): PMA standard errors, reference-free (d=10) vs. SVA (d=11)

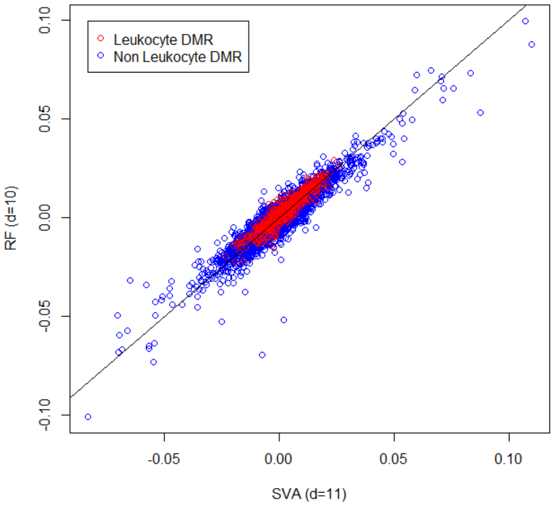

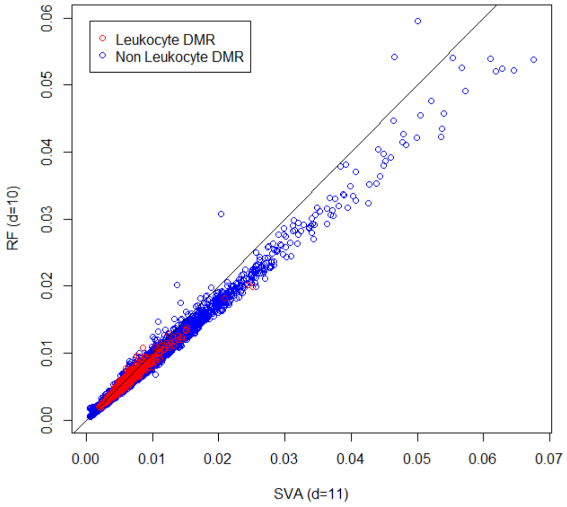

Panel (i): PMA coefficient estimates, SVA (d=11) vs. reference-adjusted (500 DMRs)

Panel (j): PMA standard errors, SVA (d=11) vs. reference-adjusted (500 DMRs)

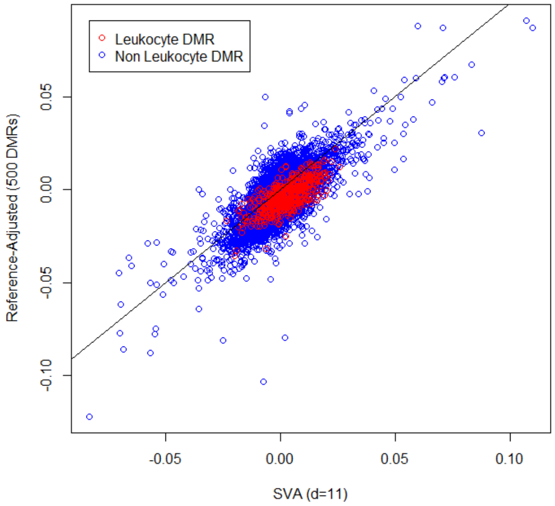

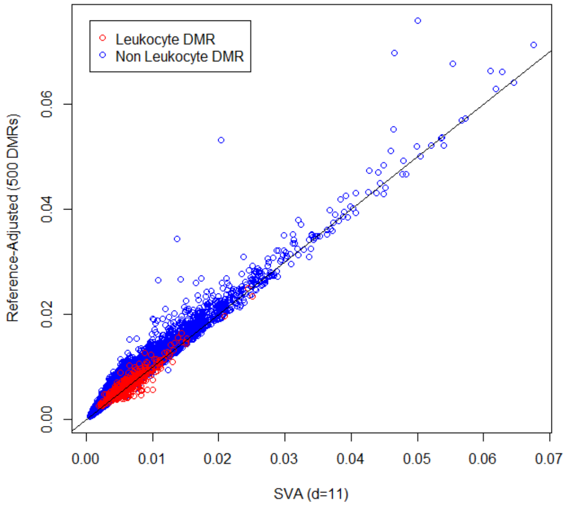

Panel (k): PMA coefficient estimates, reference-adjusted (100 DMRs) vs. reference-adjusted (500 DMRs)

Panel (l): PMA standard errors, reference-adjusted (100 DMRs) vs. reference-adjusted (500 DMRs)

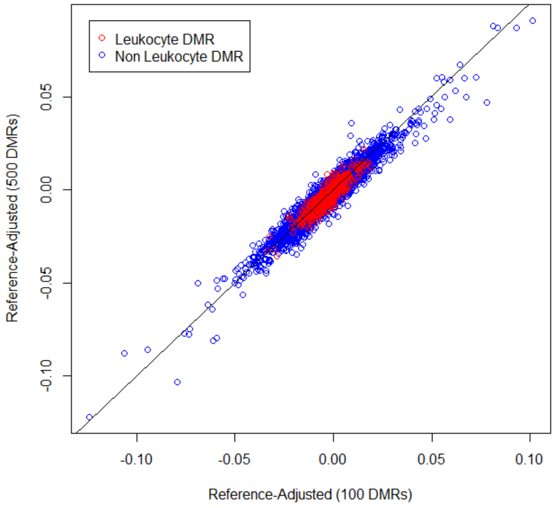

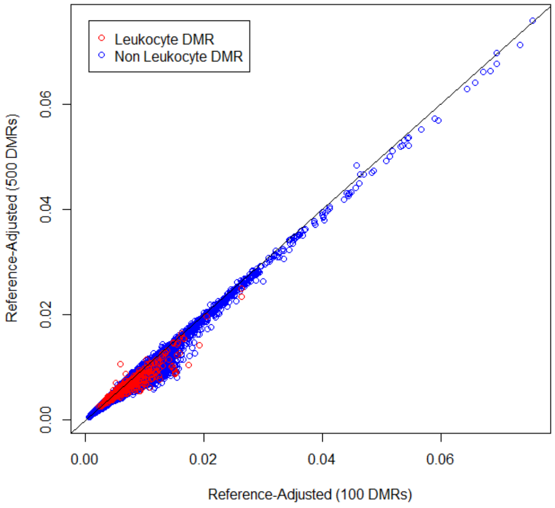

Panel (m): PMA coefficient estimates, SVA (*d*=11) vs. SVA (*d*=10)

Panel (n): PMA standard errors, SVA (*d*=11) vs. SVA (*d*=10)

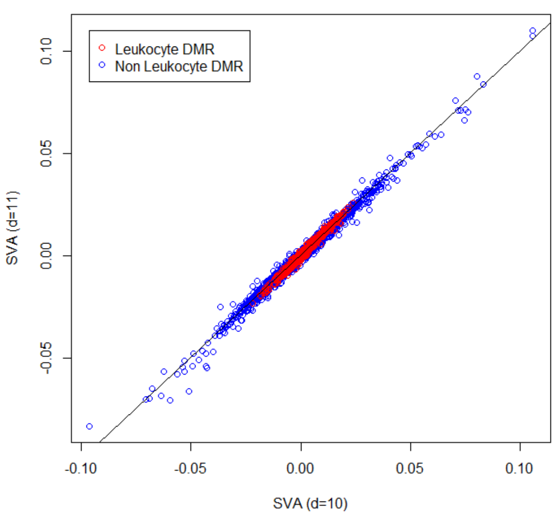

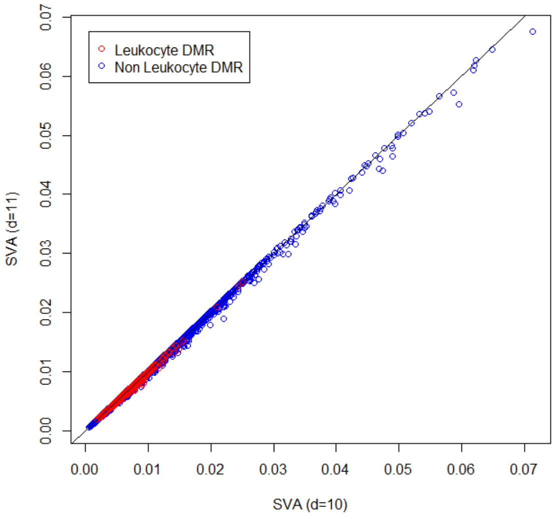

Panel (o): RMSE, comparison of reference-free and SVA to reference-based adjustments (500 DMRs)

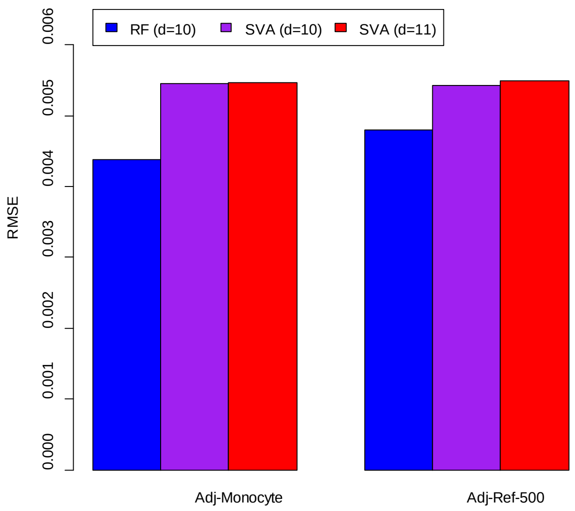

VIII. Additional details for analysis of placenta/SGA data set (Bannister et al., 2011)

Figure S11 below compares significance ($\log_{10}$ p-value) for reference-free adjusted vs. unadjusted analyses as well as for reference-free vs. SVA-adjusted analyses. Figures S11(a) and S11(c) show scatter-plots, while S11(b) and S11(d) show quantile-quantile plots. Finally, Figure S11(e) compares coefficient estimates between the reference-free and SVA approaches. Overall, the SVA and reference-free results were similar to each other and quite different from the adjusted analysis. In particular, the unadjusted analysis results in much higher apparent significance. Assuming that the results obtained by Bannister et al. (2011) were driven by cell mixtures without any direct effects (a reasonable interpretation given the results of this analysis), along with the relatively higher magnitude of microarray error (see Figure S2), we would anticipate similarity between SVA and reference-free approaches [e.g., see Figure S5(d)].

Note that the omnibus significance test described in Section V of this Supplement produced $p \approx 0.017$ for the unadjusted analysis ($\boldsymbol{\beta}_2^*$) and $p=0.20$ for the reference-free adjusted analysis ($\boldsymbol{\beta}_2$); these results suggest that DNA-methylation associations with SGA were driven by cell composition.

Figure S11: Additional comparison plots for placenta data set (Bannister et al., 2011)

Panel (a): Scatter-plot of $\log_{10}$ p-values for reference-free adjusted vs. unadjusted analysis

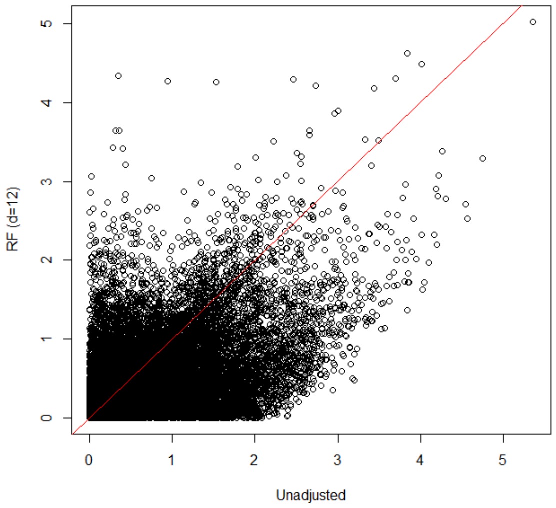

Panel (b): QQ plot of $\log_{10}$ p-values for reference-free adjusted vs. unadjusted analysis

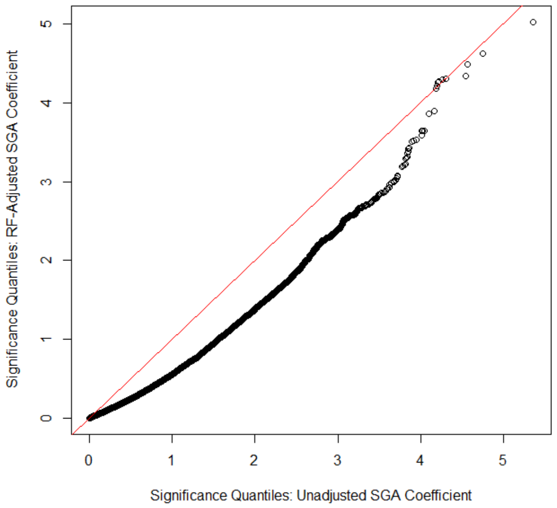

Panel (c): Scatter-plot of $\log_{10}$ p-values for reference-free adjusted vs. SVA-adjusted analysis

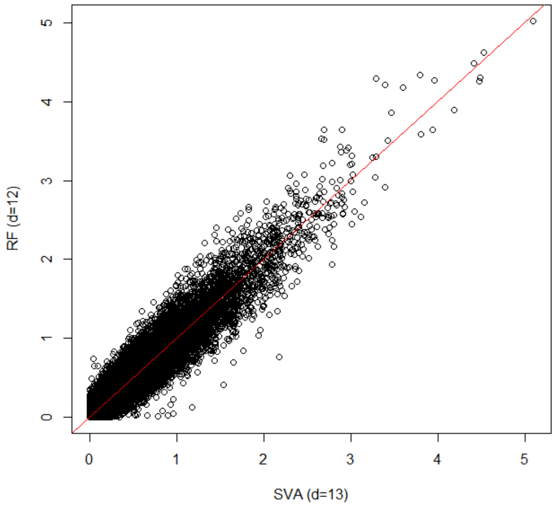

Panel (d): QQ plot of $\log_{10}$ p-values for reference-free adjusted vs. SVA-adjusted analysis

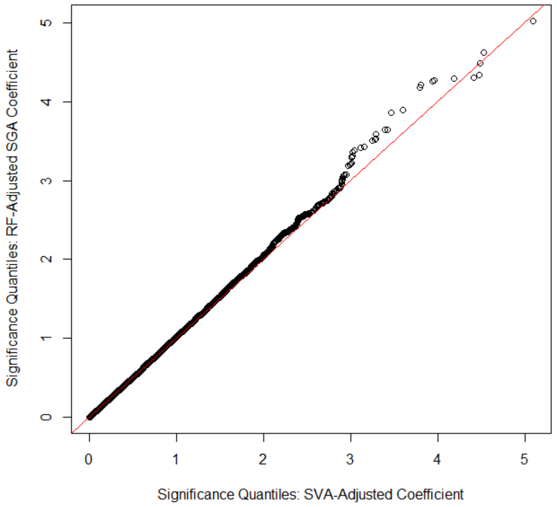

Panel (e): Scatter-plot of coefficient estimates for reference-free adjusted vs. SVA-adjusted analysis

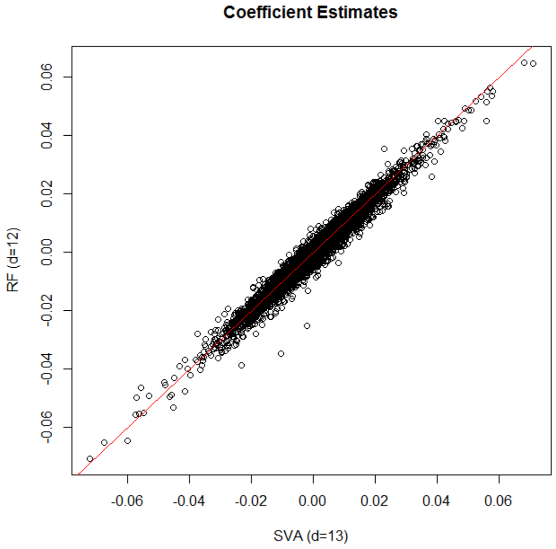

Panel (f): Scatter-plot of coefficient standard errors for reference-free adjusted vs. unadjusted analysis

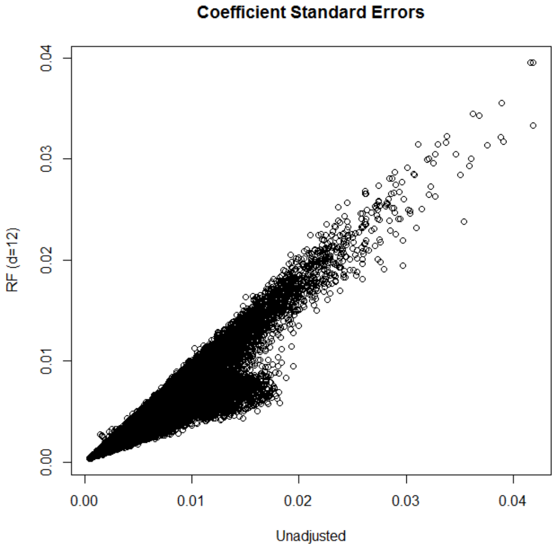

Panel (g): Scatter-plot of coefficient standard errors for SVA-adjusted analysis vs, unadjusted

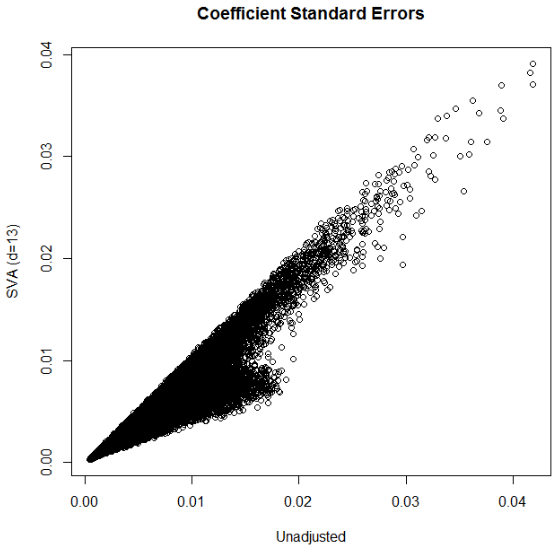

Panel (h): Scatter-plot of coefficient standard errors for reference-free adjusted vs. SVA-adjusted analysis

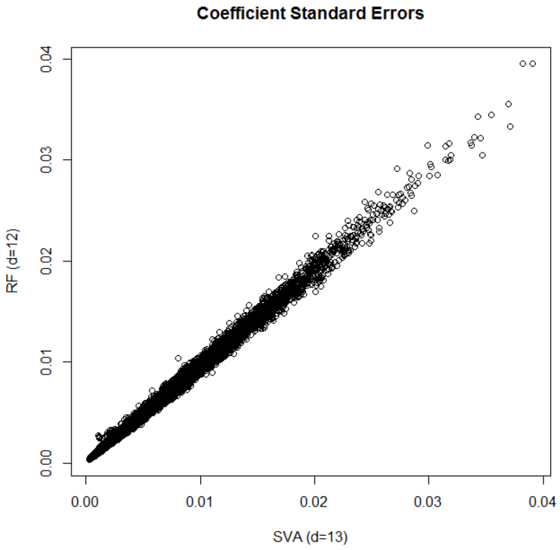

IX. Analysis of gastric cancer data set (Zouridis et al., 2012)

Our final analysis consisted of Illumina Infinium HumanMethylation27array data for 203 gastric tumors and 94 gastric non-malignant samples, originally published by Zouridis, Deng et al. (2012) and available in GEO, Accession number GSE30601. In each of three analyses of DNA methylation at autosomal CpGs, we compared tumors to non-malignant samples. The first analysis was unadjusted; the second analysis was adjusted for 27 surrogate variables [with d =27 determined by the method of Buja and Eyuboglu (1992)]; in the third, we applied the reference-free approach proposed in this article, with d = 24. Note that the RMT dimension estimation method of Teschendorff et al. (2011) produced d = 24; AIC produced d = 25, and BIC produced d = 13.

Q-values were computed for each of the three approaches using the R package *qvalue*. The unadjusted approach produced 22,211 CpG coefficients having q<0.05, the adjusted approach produced 23,959 CpG coefficients having q<0.05, and the reference-free approach produced 846 CpG coefficients with q<0.05. The estimated proportions of null CpGs were 0.17, 0.13, and 0.71 respectively. The omnibus significance test described in Section V of this Supplement produced p<0.002 for the unadjusted analysis ($\boldsymbol{\beta}_2^*$) and p≈0.008 for the reference-free ($\boldsymbol{\beta}_2$). These results, in combination with the volcano plots shown below in Figure S12(a), suggest vastly different significance levels for all three approaches, even though all three approaches result in a large number of significant CpG coefficients. Note that the difference in coefficient estimates between adjusted and reference-free analyses, and between SVA-adjusted and reference-free analyses, varied significantly by polycomb target (PcG) status of genes to which CpGs were mapped (Wilcoxon p<0.0001 for both comparisons), where polycomb status was determined by combining four known polycomb references containing gene lists of PcG-targets identified in embryonic cells (Bracken et al., 2006, Lee et al, 2006, Squazzo et al., 2006, Schlesinger et al., 2007), calling any gene a polycomb target if it appeared in any of these four references. In general, polycomb target CpGs appeared more hypomethylated in gastric cancers for the reference-free approach, compared with unadjusted and SVA-adjusted analysis.

Figure S12: Graphical analysis of results of gastric cancer data set (Zouridis et al., 2012)

Panel (a): Volcano Plots

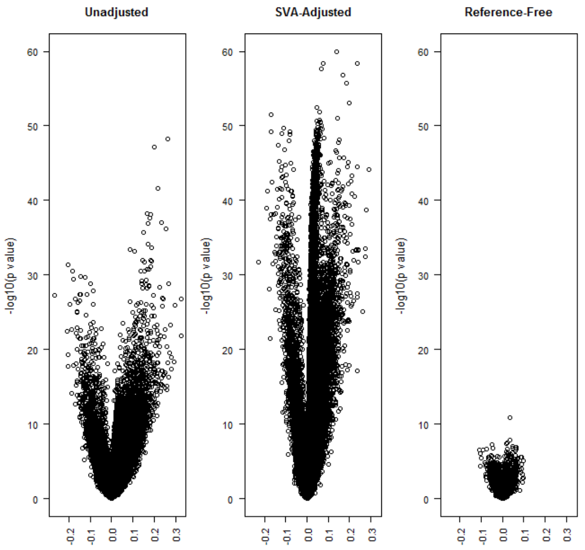

Panel (b): gastric tumor coefficient estimates, reference-free (*d*=24) vs. unadjusted

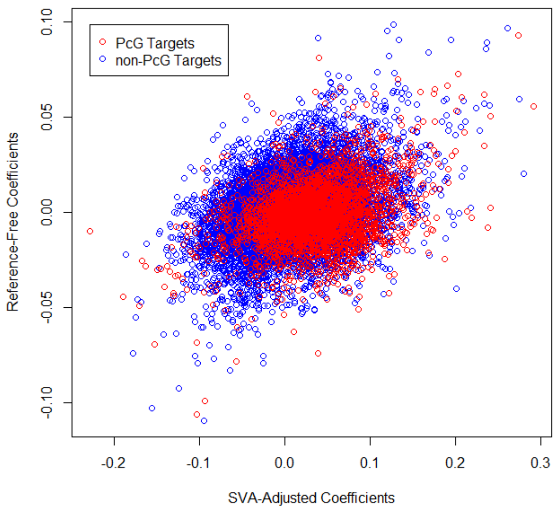

Panel (c): Difference in coefficient estimates by polycomb target status, reference-free (*d*=24) vs. unadjusted

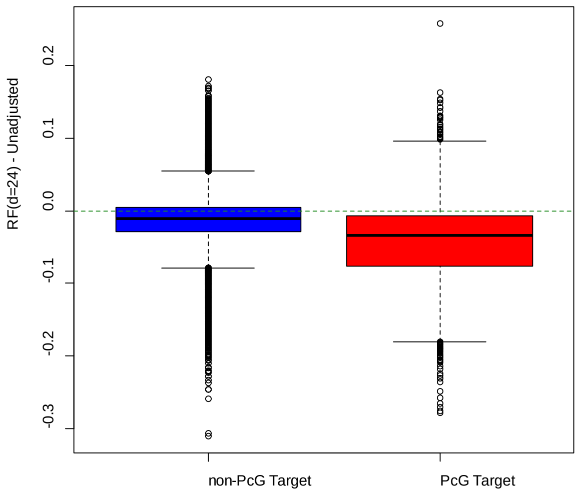

Panel (d): PMA standard errors, reference-free (*d*=10) vs. SVA (*d*=27)

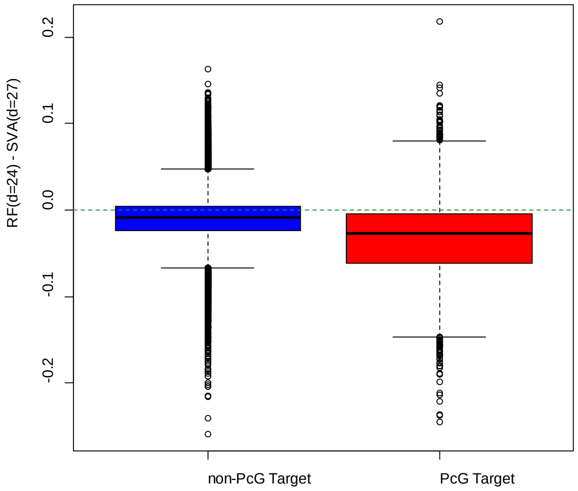

Supplement: Supplementary Data [file supp_btu029_RefFree-Houseman-Bioinformatics-R1-Supplement.pdf]
